# Harnessing natural products to target programmed cell death for intervertebral disc degeneration: Mechanisms and opportunity

**DOI:** 10.1016/j.jpha.2026.101583

**Published:** 2026-02-09

**Authors:** Songlin Liang, Zhichao Li, Renchang Chen, Shang Gao, Xintian Qu, Chenmoji Wang, Di Gao, Hao Wang, Jiatong Li, Nianhu Li

**Affiliations:** First College of Clinical Medicine, Shandong University of Traditional Chinese Medicine, Jinan, 250355, China

**Keywords:** Natural products, Programmed cell death, Intervertebral disc degeneration, Phytoconstituents

## Abstract

Intervertebral disc (IVD) degeneration (IVDD) is a common age-related degenerative disorder and a leading cause of disability and mobility impairment in adults. It is characterized by dysfunction of nucleus pulposus cells (NPCs), annulus fibrosus (AF) cells (AFCs), and cartilage endplate (CEP) cells (CEPCs), triggered by aging, inflammation, and nutrient deprivation. These cellular alterations ultimately result in structural deterioration and functional impairment of the IVD. Programmed cell death (PCD), a key regulatory mechanism governing cell fate, precisely controls survival or elimination through highly conserved and specifically activated signaling cascades. Growing evidence indicates that natural products can modulate PCD pathways and slow IVDD progression, highlighting their potential as complementary therapies or novel drug candidates for IVDD management. In light of recent advances, this review systematically summarizes the mechanisms and interactive networks of six major PCD types: apoptosis, autophagy, ferroptosis, pyroptosis, necroptosis, and PANoptosis. It further explores how natural products regulate these processes to mitigate IVDD, as well as their prospects and strategies for clinical translation. Together, these insights provide a foundation for developing natural product-based therapeutics against IVDD.

## Highlights

• Three major cell types constitute the structural and functional foundation of IVD.
• Six major PCDs are critically involved in driving IVDD pathogenesis.
• Modulation of the cell death network should focus on restoring homeostatic balance rather than generalized suppression.
• Precision targeting of PCDs based on cellular sensitivity profiles mitigates IVDD progression.
• Natural products alleviate IVDD by targeting PCD pathways.

## Graphical abstract

**Figure:**
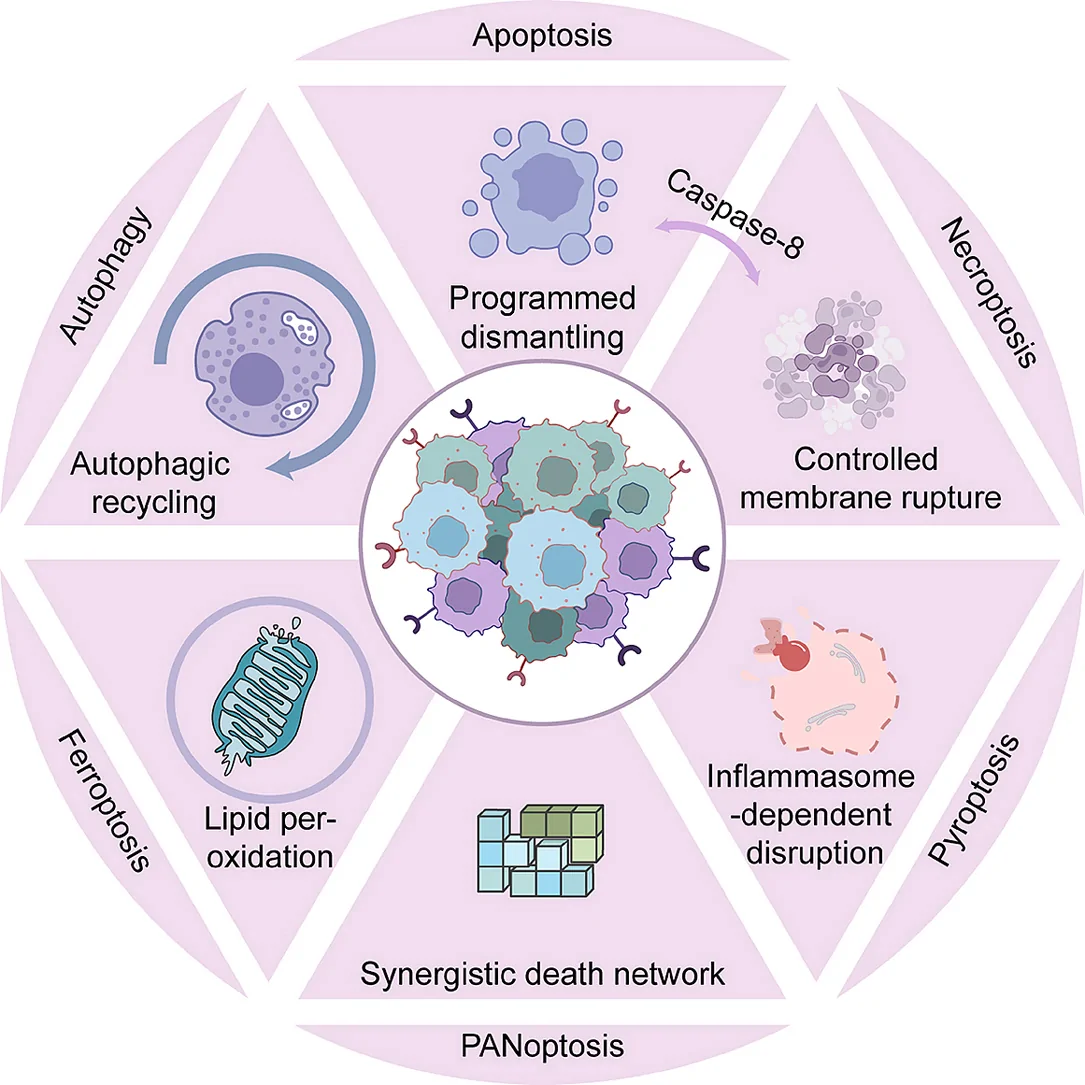

## 1 Introduction

Intervertebral disc (IVD) degeneration (IVDD) is a chronic degenerative spinal disorder primarily characterized by abnormal morphology and distribution of the nucleus pulposus (NP). Clinically, it manifests as low back pain (LBP) and, in some cases, sensory or motor dysfunction of the extremities. Globally, LBP poses a substantial burden of disability; in 2022, the total all-age disability-adjusted life years reached 71,035,000 and are projected to rise to 95,510,000 by 2050 [1]. At the individual level, nearly 80% of people experience at least one episode of LBP in their lifetime, with IVDD accounting for approximately 40% of these cases [2,3]. With the global trend of population aging, the incidence of IVDD remains persistently high and is expected to increase further [4].

The core mechanism of IVDD can be described as a multifactorial pathological process marked by progressive structural deterioration and functional impairment. Chronic inflammatory infiltration, oxidative stress imbalance, and aberrant programmed cell death (PCD) within the IVD and surrounding tissues collectively serve as major risk factors for disease onset and progression. These pathological changes initially induce metabolic dysfunction and reduced cellular activity in IVD-associated cells, leading to impaired nutrient supply and compromised biomechanical stability of the disc. As IVDD advances, cellular-level damage accumulates and evolves into tissue-level alterations, most notably a decline in load-bearing capacity accompanied by pain [5]. If these pathological factors persist without effective intervention, they can initiate irreversible degenerative cascades, ultimately disrupting disc integrity and perpetuating a vicious cycle of degeneration [6] (Fig. 1).

**Fig. 1:**
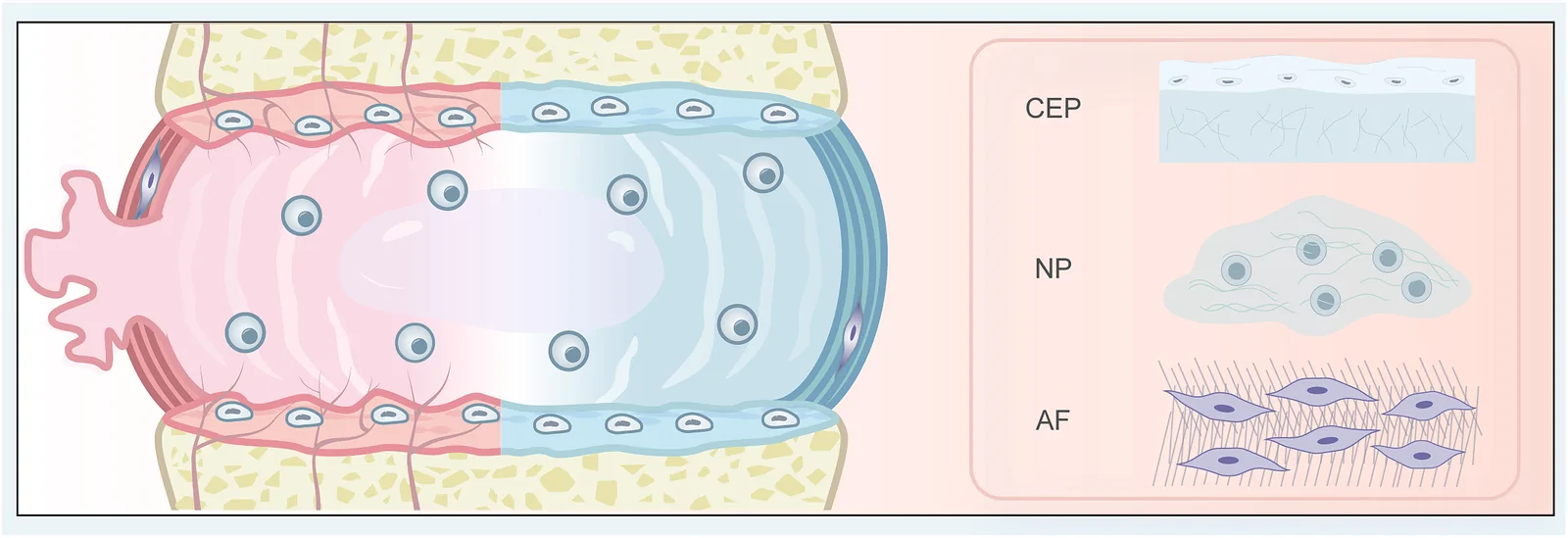
Structure of intervertebral disc (IVD). The IVD is primarily composed of the central nucleus pulposus (NP), the peripheral annulus fibrosus (AF), and the superior and inferior cartilaginous endplates (CEP).

Current therapeutic options for IVDD remain limited. Alternative therapies such as acupuncture and osteopathy are often employed in the early stages to improve patients’ quality of life, but these approaches generally provide only short-term symptomatic relief [7]. When such therapies fail to halt disease progression, nonsteroidal anti-inflammatory drugs (NSAIDs) and opioids are commonly prescribed, which may partially alleviate pain. However, these pharmacological interventions do not target the underlying pathology of IVDD and are unsuitable for long-term use due to adverse effects, including drug-related complications and the risk of addiction [8]. Surgical intervention is typically reserved for patients who do not respond to conservative treatment or who develop progressive motor and sensory deficits. Its primary aim is to relieve compression and prevent neurological dysfunction [2]. Nevertheless, early surgical intervention is not recommended as a first-line strategy, given ongoing controversies regarding its long-term effectiveness in functional recovery as well as concerns about its high cost [9]. Considering these limitations, there is an urgent need for novel and effective pharmacological interventions to alleviate IVDD symptoms and address its underlying mechanisms. The development of such therapeutics, however, requires deeper exploration of disease-specific molecular and cellular pathways.

PCD, a critical regulatory mechanism of cellular physiology, is an active process in which cells undergo controlled elimination upon receiving specific signals to maintain homeostasis during development. This process plays an essential role in removing senescent or damaged cells, thereby preserving normal tissue metabolism [10]. Dysregulation of PCD is a key contributor to microenvironmental metabolic disturbances, and persistent aberrant PCD represents a common pathological hallmark of tissue degeneration. The proper function of the IVD relies on its highly organized cellular architecture, and dysregulated PCD in any cellular component can lead to IVD dysfunction. This mechanistic insight suggests that targeted modulation of PCD in distinct IVD cell populations could serve as a promising therapeutic strategy to effectively slow or reverse IVDD progression. To date, six major forms of PCD have been implicated in IVDD pathogenesis: apoptosis, autophagy, ferroptosis, pyroptosis, necroptosis, and PANoptosis. Each of these modes of cell death exhibits distinct molecular mechanisms and characteristics, yet all ultimately contribute to IVD cellular damage. Importantly, the preferential activation of specific PCD types in different IVD cell populations and their varying contributions to disease progression highlight the heterogeneity of IVDD pathogenesis. Systematic elucidation of these differences and precise characterization of PCD roles in IVDD will deepen mechanistic understanding of the disease and provide a theoretical basis for developing cell type-specific therapeutic strategies [6].

With the advancement of pharmacological research, natural products have attracted increasing attention due to their unique advantages. Several have shown potential in targeting and modulating PCD in IVD-related cells, thereby alleviating the progression of IVDD. Owing to their relatively low cost, defined bioactive components, and reduced adverse effects, natural products represent promising alternatives to existing drugs and are emerging as a major focus in novel drug development [11]. This review highlights six major PCD types implicated in IVDD research: apoptosis, autophagy, ferroptosis, pyroptosis, necroptosis, and PANoptosis. We systematically summarize recent advances in natural product-mediated regulation of these PCD pathways in IVD-related cells (Supplementary data and Fig. S1), elucidate their mechanisms and characteristics during IVDD progression, and examine their potential roles in disease pathogenesis. Furthermore, we aim to provide new insights and references for future research and clinical translation of natural products targeting PCDs as therapeutic strategies against IVDD.

## 2 Cellular composition of the IVD

The major cellular components of a normal IVD include four cell types: NP cells (NPCs), cartilage endplate (CEP) cells (CEPCs), annulus fibrosus (AF) cells (AFCs), and notochordal cells (NCs). Notably, NCs disappear completely during childhood, typically before the age of 10, and are therefore not considered to play a pivotal role in the pathogenesis of IVDD [12]. For this reason, NCs are excluded from the present discussion.

NPCs are among the most functionally important cell types in the IVD and constitute the predominant cellular population in the adult NP. Derived from mesenchymal stem cells (MSCs) induced by NCs, NPCs actively secrete large extracellular matrix (ECM) macromolecules, particularly glycosaminoglycans. These ECM components not only give the NP its characteristic gelatinous appearance but also impart hydrophilic and elastic properties that enable it to withstand and dissipate both intrinsic and extrinsic mechanical stresses [13].

AFCs are the principal functional cells of the AF and play a crucial role in maintaining the structural stability of the IVD. They are systematically distributed across 15–25 concentrically arranged collagenous lamellae, where they continuously secrete a collagen type I-rich ECM [14]. This secretory activity forms the characteristic lamellar architecture of the AF, which stabilizes the position of the NP and helps restrict excessive spinal motion [15]. Functionally complementary to NPCs, AFCs contribute to the disc's tensile strength and stress resistance, while NPCs maintain compressive elasticity, together ensuring the comprehensive functionality of the IVD.

CEPCs are predominantly arranged in columnar arrays within the lacunae of the CEP. Their ECM, enriched in collagen type II (COL2) and aggrecan, maintains the elasticity and compressibility of the CEP while also regulating nutrient transport [16]. The AF and CEP form close structural connections that establish the relatively sealed architecture of the IVD. Together with immunosuppressive molecular factors, they function as a blood-NP barrier, effectively isolating the NP from the host immune system and thereby preserving homeostasis within the IVD microenvironment [17].

Aberrant PCD in all three major cell types contributes to the development of IVDD, albeit through distinct mechanisms. The impairment of NPCs and subsequent NP degeneration constitute the most fundamental pathological features of IVDD. In contrast, damage to the AF and CEP does not directly initiate IVDD but instead alters mechanical loading of the disc and disrupts nutrient and metabolic waste transport. These changes lead to metabolic dysfunction in NPCs and progressive NP degradation, the hallmark of degeneration. Ultimately, the compromised AF or CEP may allow NP herniation, giving rise to the characteristic symptoms of IVDD, including pain, motor dysfunction, and sensory abnormalities [15].

## 3 Natural products regulate PCDs for IVDD

### 3.1 Apoptosis

#### 3.1.1 Overview of apoptosis

Apoptosis, the most common form of PCD, is characterized by cellular rounding, chromatin condensation within the nucleus, and membrane blebbing that produces apoptotic bodies. Importantly, the integrity of the plasma membrane is preserved, thereby preventing inflammation. This process is mediated by caspases, which exist as inactive zymogens and are classified as initiators or effectors. Initiator caspases are activated in response to pro-apoptotic signals such as interleukins (ILs), tumor necrosis factor-α (TNF-α), and endoplasmic reticulum (ER) stress, whereas effector caspases dismantle the cell once apoptosis is initiated. Certain caspases also serve as molecular links between apoptosis and other forms of PCD, without directly executing cell death [18].

Apoptosis proceeds through three major pathways. The intrinsic (mitochondrial) pathway is triggered by oxidative stress, DNA damage, or elevated intracellular calcium. These stimuli activate pro-apoptotic proteins such as B-cell lymphoma-2 (Bcl-2)-associated K protein and Bcl-2-associated X protein (BAX), which induce mitochondrial outer membrane permeabilization or mitochondrial permeability transition pore opening. This event promotes the release of cytochrome c (Cyto C), second mitochondria-derived activator of caspases (Smac/DIABLO), and apoptosis-inducing factor (AIF) into the cytosol [19]. Cyto C associates with pro-caspase-9 and apoptotic protease-activating factor-1 (APAF-1) in the presence of adenosine triphosphate (ATP)/deoxyadenosine triphosphate (dATP) to form the apoptosome, which subsequently activates the caspase cascade leading to apoptosis [20]. The extrinsic (death receptor) pathway is initiated when extracellular ligands bind to transmembrane death receptors containing death domains. For example, TNF-α binding to its receptor TNF receptor 1 (TNFR1) recruits caspase-8, which activates caspase-3 and forms death-inducing signaling complexes (DISCs), thereby initiating apoptosis. Caspase-8 plays critical roles in both intrinsic and extrinsic pathways. In the intrinsic pathway, it helps activate downstream effector caspases (i.e., caspase-3, caspase-6, and caspase-7). In the extrinsic pathway, TNFR engagement recruits adaptor proteins including TNFR-associated factors, receptor-interacting protein (RIP) kinases (RIPKs), and inhibitors of apoptosis proteins to assemble complex I, which can transition into either complex IIA (TNFR1-associated death domain protein (TRADD)-Fas-associated death domain (FADD)-caspase-8) or complex IIB (RIPK1-FADD-caspase-8), both of which activate caspase-8 and drive apoptosis [21].

ER stress-induced apoptosis is stimulus-specific and is typically triggered by the accumulation of misfolded proteins. Initially, the unfolded protein response (UPR) acts to restore proteostasis; however, when ER stress is prolonged or severe, the UPR shifts from a protective to a pro-apoptotic program. This transition is mediated through the three canonical UPR branches: inositol-requiring enzyme 1α, protein kinase R-like ER kinase, and activating transcription factor 6 (ATF6). These signaling pathways converge on CCAAT/enhancer binding protein (C/EBP) homologous protein (CHOP), which integrates proteotoxic stress into apoptotic signaling cascades, thereby committing the cell to apoptosis [22,23] (Fig. 2A).

**Fig. 2:**
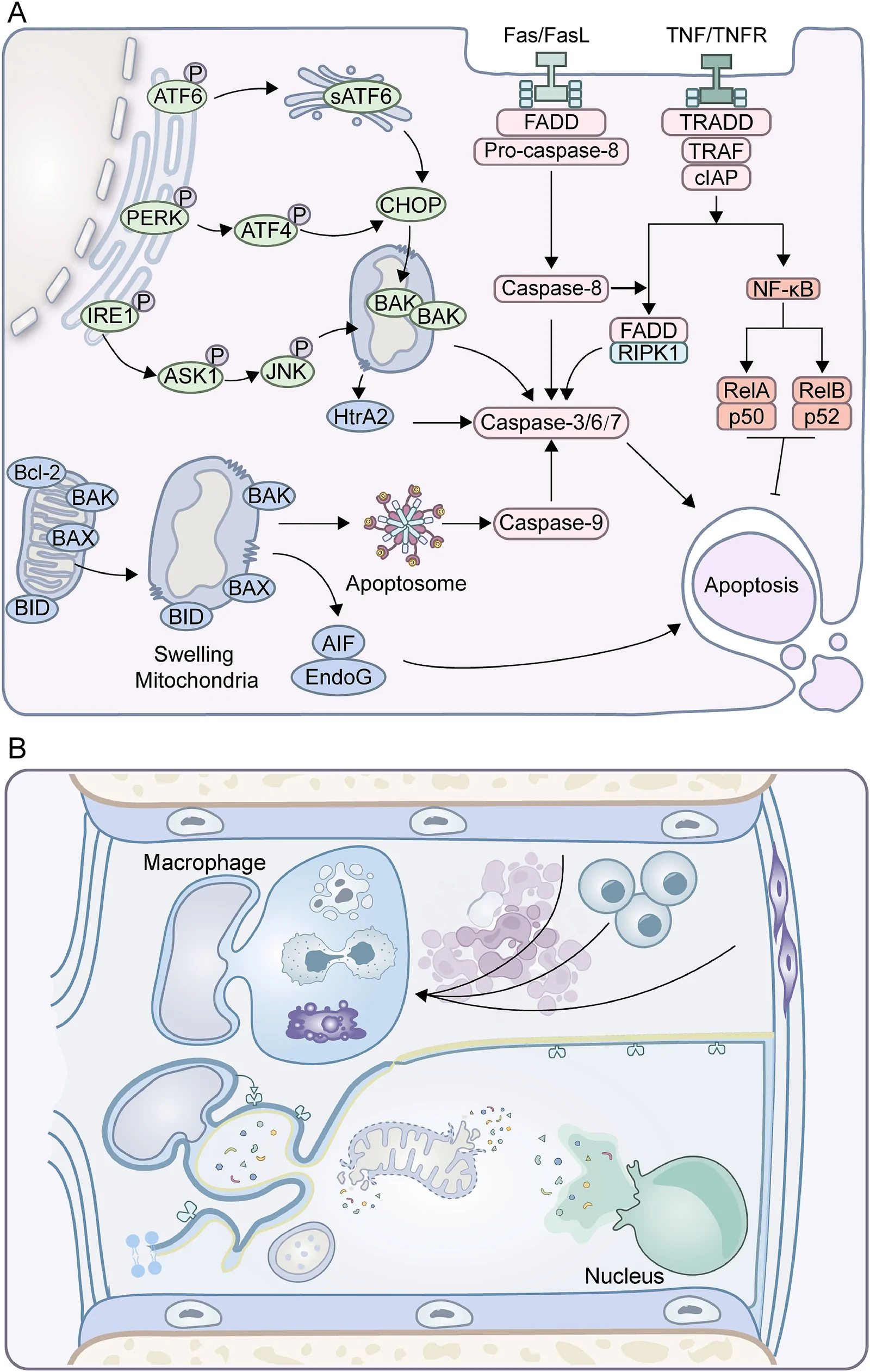
Mechanisms of apoptosis in intervertebral disc (IVD) degeneration (IVDD). (A) Apoptosis proceeds through three principal pathways: the intrinsic (mitochondrial) pathway regulated by B-cell lymphoma 2 (Bcl-2) family proteins, the extrinsic (death receptor) pathway mediated by death ligand/receptor interactions, and endoplasmic reticulum (ER) stress-induced apoptosis triggered by proteotoxic stress. (B) Excessive apoptosis of IVD cells, marked by “eat-me” signals, results in tissue loss, followed by macrophage-mediated clearance of the cellular debris. ATF6: activating transcription factor 6; sATF6: short ATF6; Fas: Fas receptor; FasL: Fas ligand; TNF: tumor necrosis factor; TNFR: TNF receptor; FADD: Fas-associated death domain; TRADD: TNFR1-associated death domain protein; TRAF: TNFR-related factor; cIAP: cellular inhibitor of apoptosis protein; PERK: protein kinase R-like ER kinase; CHOP: CCAAT/enhancer binding protein (C/EBP) homologous protein; IRE1: inositol requiring protein-1; ASK1: apoptosis signal-regulating kinase 1; JNK: c-Jun N-terminal kinase; BAK: Bcl-2 associated K protein; RIPK: receptor-interacting protein (RIP) kinase; NF-κB: nuclear factor kappaB; HtrA2: HtrA serine peptidase 2; RelA: v-rel reticuloendotheliosis viral oncogene homolog A; BAX: Bcl-2-associated X protein; BID: BH3-interacting domain death agonist; AIF: apoptosis-inducing factor; EndoG: endonuclease G.

#### 3.1.2 Apoptosis in IVDD

Apoptosis plays a fundamental role in regulating biological processes by modulating cell cycle progression, thereby maintaining tissue homeostasis and metabolic stability. Physiologically appropriate levels of apoptosis are therefore essential for sustaining IVD health [24]. However, pathological states often arise from “suboptimal intensity” of biological processes. The IVD, being a hypoxic and avascular tissue, exhibits slower cellular turnover and reduced metabolic activity compared with most other tissues. In addition, its critical roles in weight-bearing and shock absorption subject IVD cells to persistent mechanical stress. These unique features render IVD cells highly vulnerable to damage, less capable of recovery, and more prone to excessive apoptotic activation [25]. In the pathogenesis of IVDD, three fundamental pathological phenomena are commonly observed: cellular dysfunction, widespread ECM degradation, and inflammatory responses. As the predominant form of PCD in NPCs, apoptosis directly contributes to cellular dysfunction and ECM degradation, while indirectly amplifying inflammatory signaling [26]. Furthermore, apoptosis of AFCs and CEPCs leads to structural damage in their respective tissues, which in turn exacerbates NPC apoptosis and accelerates IVDD progression.

Apoptosis-related changes are frequently observed during the progression of IVDD. Patient-derived IVD samples show significant upregulation of apoptotic markers such as Fas receptor, Fas ligand, and death receptor 4 compared with normal controls [27]. Similarly, the expression intensity of the TNF-α/TNFR signaling axis correlates strongly with apoptosis levels in NPCs [28]. Abnormal mechanical stress, one of the most recognized risk factors for IVDD and a widely used experimental modeling approach, has been shown to markedly increase apoptosis in both NPCs and CEPCs across various *in vivo* and *in vitro* studies [29]. In addition, age-related tissue senescence, nutrient deprivation, and estrogen deficiency contribute to IVDD progression by inducing ER stress and mitochondrial damage [30].

As noted earlier, excessive apoptosis is a consistent feature of IVDD, making apoptosis inhibition a direct therapeutic strategy. Experimental evidence supports this approach: targeted silencing of the mechanosensitive protein Piezo1 has been shown to protect IVD tissue by reducing apoptosis in AFCs [31]. In addition, nanoparticle-mediated delivery of miR-150-5p to NPCs effectively alleviates both puncture-induced and inflammation-associated apoptosis, thereby mitigating IVDD progression. Mechanistically, this occurs through regulation of F-box and WD repeat domain-containing 11 (FBXW11) and transforming growth factor-β (TGF-β)-activated kinase 1 (TAK1), ultimately suppressing apoptosis in experimental models [32]. Collectively, these findings validate apoptosis suppression as a promising and reliable strategy for IVDD drug development (Fig. 2B).

#### 3.1.3 Natural products alleviate IVDD by targeting apoptosis

Apoptosis inhibition represents an established therapeutic approach for IVDD, with numerous natural products demonstrating regulatory effects on apoptotic states in NPCs, AFCs, and CEPCs, thereby serving as potential apoptosis inhibitors for IVDD intervention that warrant either clinical application or further mechanistic investigation. We have summarized the natural products regulating apoptosis in IVD-related cells and systematically classified their research methodologies and specific mechanisms of action (Table S1).

### 3.2 Autophagy

#### 3.2.1 Overview of autophagy

Autophagy is an evolutionarily conserved eukaryotic process that degrades damaged organelles, misfolded proteins, and pathogens under pathological conditions to recycle substrates and maintain cellular homeostasis. Unlike apoptosis, autophagy is characterized by four distinctive features: i) self-digestion, the reutilization of intracellular components; ii) rapidity, completion typically occurs within 3 h of initiation; iii) non-specificity, which process lacks strict target selectivity; and iv) ubiquity, which occurs across diverse cell types and species, including tumor cells that maintain basal autophagic flux [33].

Autophagy is classified into three types based on substrate delivery to lysosomes: macroautophagy, microautophagy, and chaperone-mediated autophagy. Among these, macroautophagy, the most extensively studied form, is commonly referred to simply as autophagy and will be the focus of this review. This evolutionarily conserved and tightly regulated process involves multiple autophagy-related proteins (ATGs) and progresses through four sequential phases: initiation, elongation, maturation, and recycling [34].

Autophagy initiation is primarily regulated by the mechanistic target of rapamycin complex 1 (mTORC1), which interacts with the autophagy regulatory UNC-51-like kinase 1 (ULK1) complex composed of UNC-51, ATG13, focal adhesion kinase family interacting protein of 200 kD (FIP200), and ATG101 [35]. Under nutrient-rich conditions, mTORC1 suppresses autophagy by phosphorylating ULK1 and ATG13. During starvation, inhibition of mTORC1 releases the ULK1 complex, which translocates to the ER and activates the Beclin-1 (BECN1)- phosphatidylinositol 3-kinase (PI3K) catalytic subunit type 3/vacuolar protein sorting 34 (PIK3C3/VPS34) complex. This activation promotes the generation of phosphatidylinositol-3-phosphate, initiating phagophore formation, with recruitment of ATG12 and ATG8 family proteins [36]. The ATG12-ATG5-ATG16L complex is formed through ATG10-mediated conjugation, while members of the ATG8 family, including microtubule-associated protein 1 light chain 3 (LC3), are conjugated to phosphatidylethanolamine (PE) on the phagophore membrane. This lipidation converts LC3-I into LC3-II, a widely recognized marker of autophagy. The phagophore subsequently expands into a double-membrane autophagosome, which ultimately fuses with perinuclear lysosomes for cargo degradation and recycling [37] (Fig. 3A).

**Fig. 3:**
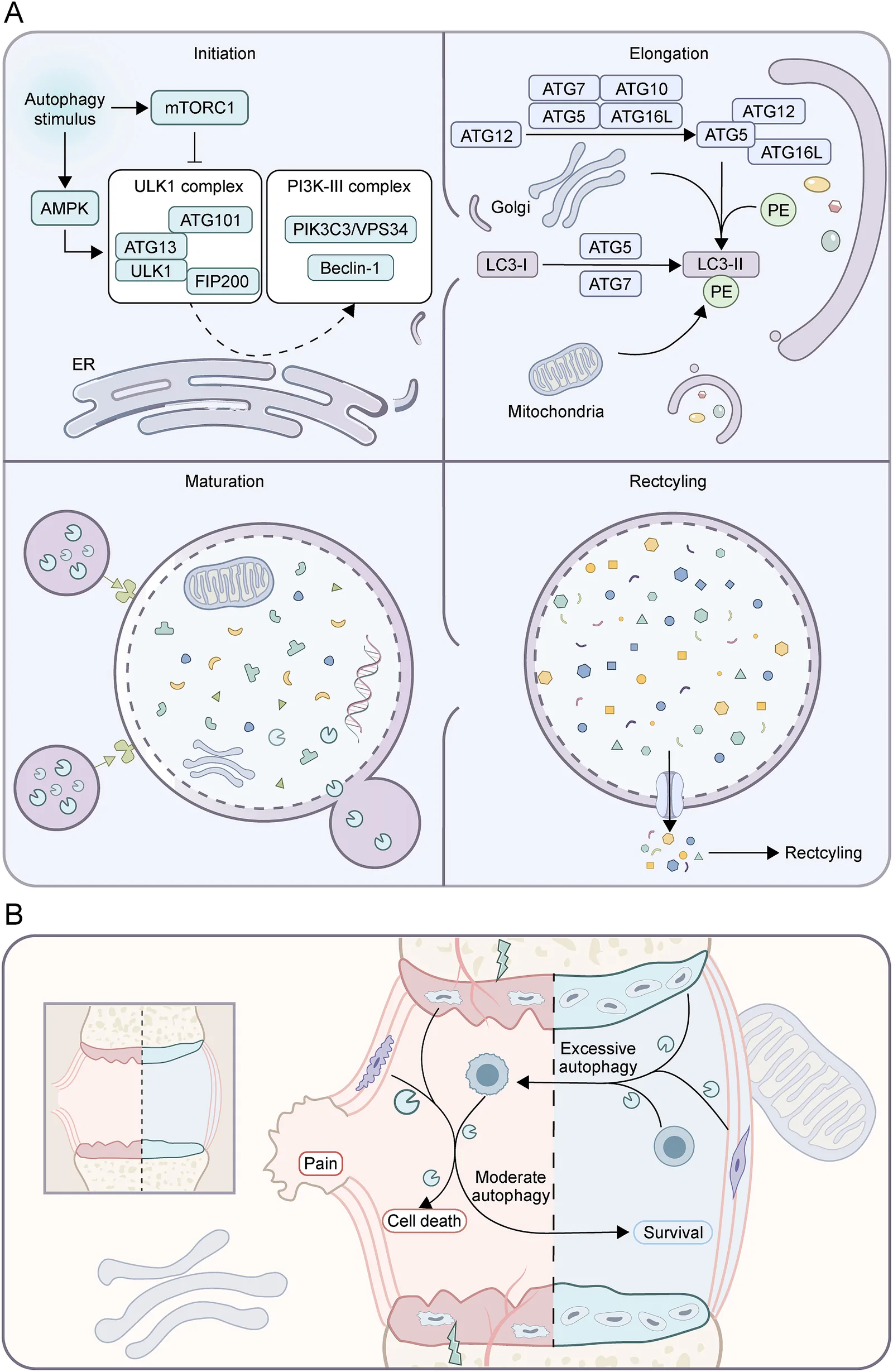
Mechanisms of autophagy in intervertebral disc (IVD) degeneration (IVDD). (A) Autophagy, induced by stimuli like nutrient deprivation, forms double-membrane autophagosomes that engulf cytoplasmic cargo, followed by lysosomal fusion enabling degradation and recycling of materials to sustain homeostasis. (B) In IVDD, dysregulated autophagy exhibits dual roles: excessive flux leads to cell death, while moderate activity maintains IVD function by clearing damaged organelles and alleviating metabolic stress. mTORC1: mechanistic target of rapamycin complex 1; AMPK: 5′-adenosine monophosphate (AMP)-activated protein kinase; ULK1: UNC-51-like kinase 1; ATG: autophagy-related proteins; FIP200: focal adhesion kinase family interacting protein of 200 kD; PI3K-III: class III phosphatidylinositol 3-kinase; PIK3C3/VPS34: PI3K catalytic subunit type 3/vacuolar protein sorting 34; ER: endoplasmic reticulum; LC3: microtubule-associated protein 1 light chain 3; PE: phosphatidylethanolamine.

#### 3.2.2 Autophagy in IVDD

Autophagy is ubiquitously present across diverse cell types, where it plays a central role in maintaining IVD homeostasis [38]. In patients with IVDD, increased autophagic flux and its associated protective effects are particularly evident in the NP. Autophagy is believed to suppress senescence in damaged NPCs, thereby mitigating disease progression [39]. During early IVD development, however, dysregulated autophagy contributes to the disappearance of NCs and IVDD-like degeneration. Conversely, regulated autophagy has been proposed to slow NC loss and preserve microenvironmental stability by restraining apoptosis [40], highlighting its antagonistic relationship with apoptotic pathways. Beyond these protective functions, autophagy also mediates the detrimental effects of abnormal mechanical stress, a well-established pathogenic factor in IVDD. For instance, mechanically overloaded degenerative IVDs exhibit elevated expression of caspase, LC3, BECN1, and CHOP, as well as enhanced lysosomal activity. These molecular alterations coincide with increased reactive oxygen species (ROS) accumulation, augmented apoptosis, and progressive IVDD aggravation in rat models [41].

NPCs are highly dependent on autophagy. As terminally differentiated cells, they rely extensively on the ER-Golgi system to sustain glycosaminoglycan synthesis [42]. Basal autophagy supports the efficiency of this secretory pathway by alleviating the UPR or through selective ER-phagy. When autophagy is impaired, persistent UPR activation occurs, which subsequently triggers apoptosis via CHOP-dependent ER stress. In addition, autophagic degradation products supply critical metabolic substrates, enabling NPCs to adapt their energy metabolism under hypoxic conditions [43].

AFCs exhibit a strong dependence on autophagy. Under nutrient deprivation, autophagic flux is directly upregulated in AFCs, reflecting an adaptive response to the nutrient-deficient microenvironment of the IVD. Thus, compared with NPCs and CEPCs, maintaining proper autophagy levels may play an even more critical role in AFC homeostasis [44]. Transcription factor EB-driven autophagy activation alleviates ER stress-mediated ECM catabolism and restores normal AFC morphology in the IVD [45]. Similarly, inhibition of mTORC1 enhances autophagy through protein kinase B (AKT) activation, reducing inflammatory responses and restoring homeostatic balance in rabbit AFCs [46]. Although mediated by distinct signaling cascades, these pathways provide comparable protective effects. Collectively, these findings highlight the role of Sirtuin (SIRT) family proteins in modulating key pathological processes in IVD, including cellular senescence, inflammation, and autophagy, suggesting that SIRTs and their autophagy-regulatory network may represent promising therapeutic targets for preserving AFC function and delaying IVD degeneration [47].

CEPCs rely on autophagy to maintain cellular homeostasis. Conditional deletion of all forkhead box O (FOXO) isoforms in the IVD using *Col2a1Cre* and *AcanCreER* mice led to spontaneous IVD degeneration, partly due to severe cell loss in both the CEP and NP. In this context, FOXO-regulated autophagy protects CEPCs from hypoxia-induced damage [48]. Importantly, autophagy regulation in CEPCs arises from distinct sources: SIRT3-mediated mitophagy preserves the chondrogenic phenotype by restoring COL2 and SRY-box transcription factor 9 expression [49], whereas SIRT1 upregulation enhances autophagosome formation and counteracts CEP calcification [50]. These findings suggest that therapeutic strategies should target specific autophagy-related pathways depending on the pathological features of IVDD (Fig. 3B).

#### 3.2.3 Natural products alleviate IVDD by targeting autophagy

Given the widespread activation of autophagy in IVDD, modulating autophagic activity, typically through its upregulation, represents a crucial research direction for protecting IVD-associated cells and mitigating disease progression [51].

Numerous natural products exert protective effects on IVD through autophagy-mediated mechanisms. For instance, naringin, derived from citrus fruits, enhances autophagic activity by promoting SIRT protein expression, reducing the inflammatory factor transcription and optimizing mitochondrial respiratory function, thereby alleviating inflammatory responses in CEPCs [49]. Conversely, sesamin upregulates *BECN2* expression to suppress excessive autophagy-induced CEPCs degeneration and associated IVD nutritional deficiency and dysfunction [52]. Pinocembrin mitigates oxidative stress-induced apoptosis and degeneration, potentially through nuclear factor erythroid 2-related factor 2 (Nrf2) activation [53]. The polyphenolic compound curcumin (Cur), derived from turmeric, attenuates mechanical stress-induced CEP damage and ECM degradation by enhancing autophagic flux [54]. These protective effects of natural products appear to depend, at least partially, on maintaining autophagic activity to monitor and selectively degrade damaged mitochondria. This biological process reflects the beneficial function of mitochondrial quality control systems, ultimately promoting IVD rejuvenation [51] (Table S2).

### 3.3 Ferroptosis

#### 3.3.1 Overview of ferroptosis

Ferroptosis is an iron-dependent form of regulated cell death, first described in 2012 during studies on the compound erastin. Unlike apoptosis and autophagy, erastin-induced cell death cannot be reversed by conventional cell death inhibitors but can be prevented through iron chelation or reduced iron uptake, highlighting its defining iron-dependent nature [55].

Ferroptosis is regulated by multiple interconnected pathways, including iron homeostasis, polyunsaturated fatty acid (PUFA) metabolism, glutathione (GSH) biosynthesis, and redox balance. Its key pathological features are toxic free iron accumulation, GSH depletion, and lipid peroxidation-induced membrane damage. Morphologically, ferroptotic cells detach from substrates, shrink, and adopt a rounded shape, accompanied by distinct mitochondrial alterations such as reduced size, loss of cristae, increased membrane density, and outer membrane rupture. Importantly, both the nuclear and plasma membranes remain intact [56].

Iron overload is a key driver of ferroptosis. Extracellular Fe^3+^ binds transferrin (Tf) and enters cells via Tf receptor 1 (TfR1)-mediated endocytosis. Within endosomes, Fe^3+^ is reduced to Fe^2+^ by six-transmembrane epithelial antigen of prostate 3 (STEAP3) and subsequently released into the labile iron pool (LIP) through divalent metal transporter 1 (DMT1). Excess Fe^2+^ can be stored in ferritin or exported by ferroportin (FPN); however, disrupted iron homeostasis leads to Fe^2+^ accumulation in the LIP. This excess iron promotes Fenton reactions, generating ROS that oxidize PUFAs, initiating lipid radical formation and membrane destabilization. Enzymes such as acyl-CoA synthetase long-chain family member 4 (ACSL4) and lysophosphatidylcholine acyltransferase 3 (LPCAT3), which facilitate PUFA incorporation into membranes, together with lipoxygenase (LOX)-mediated peroxidation, further amplify lipid damage. The combined activity of these pathways critically compromises membrane integrity, driving ferroptotic cell death [57].

Mitochondria exert dual and opposing roles in ferroptosis. On one hand, respiratory ROS production accelerates lipid peroxidation; on the other hand, mitochondrial GSH peroxidase 4 (GPX4) mitigates membrane damage by detoxifying lipid peroxides. In addition, the mitochondrial mevalonate pathway connects cholesterol metabolism to ferroptosis resistance by supporting coenzyme Q10 (CoQ10) biosynthesis, a key lipid-soluble antioxidant [58].

The cystine-glutamate antiporter (System Xc^−^)/GSH/GPX4 axis represents the central defense mechanism against ferroptosis. System Xc^−^ imports cystine for GSH synthesis, and GSH, in turn, enables GPX4 to reduce toxic lipid hydroperoxides into inert lipid alcohols [59]. In parallel, several complementary pathways provide additional protection: ferroptosis suppressor protein 1 (FSP1) reduces CoQ10, thereby quenching lipid radicals; mitochondrial dihydroorotate dehydrogenase eliminates mitochondrial lipid peroxides; and guanosine triphosphate (GTP) cyclohydrolase 1-derived tetrahydrobiopterin (BH4) acts as a radical scavenger while suppressing LOX activity [60]. Collectively, these antioxidant systems highlight the therapeutic promise of targeting ferroptosis through GPX4 activators, iron chelators, or lipid radical scavengers (Fig. 4A).

**Fig. 4:**
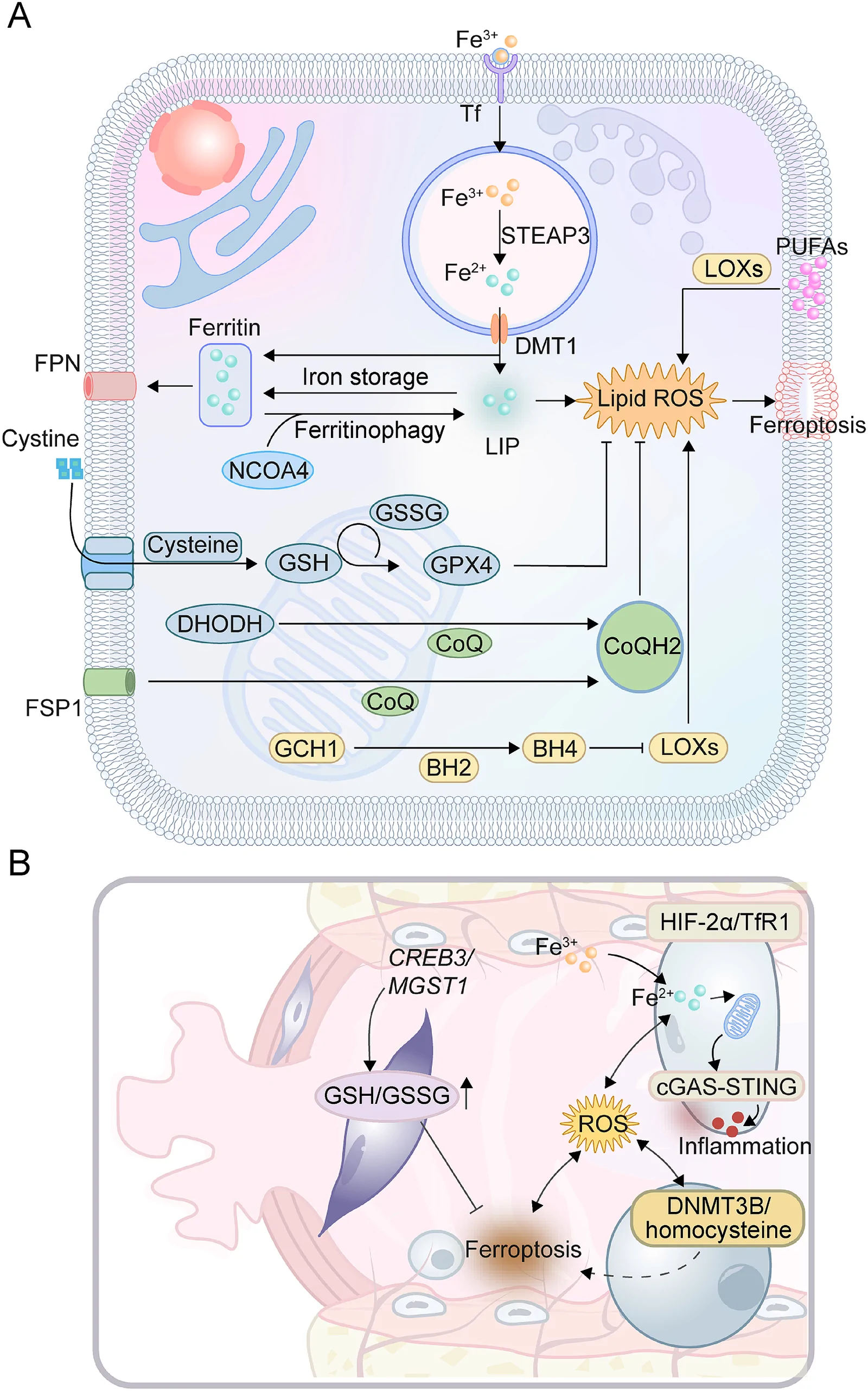
Mechanisms of ferroptosis in intervertebral disc (IVD) degeneration (IVDD). (A) Ferroptosis is an iron-dependent cell death driven by lipid peroxidation, counteracted by antioxidant systems including cystine-glutamate antiporter (System Xc^−^)/glutathione (GSH) peroxidase 4 (GPX4)/GSH and ferroptosis suppressor protein 1 (FSP1)/coenzyme Q (CoQ) pathways. (B) In IVDD, cell-type-specific regulation occurs where *CREB3*, *MGST1*, and DNA methyltransferase 3B (DNMT3B) suppress ferroptosis in annulus fibrosus (AF) cells (AFCs) and nucleus pulposus (NP) cells (NPCs), while cartilage endplate (CEP) cells (CEPCs) exhibit the cyclic guanosine monophosphate-adenosine monophosphate (GMP-AMP) synthase-stimulator of interferon (IFN) genes (cGAS-STING) pathway-mediated ferroptosis linking to inflammatory activation. Tf: transferrin; STEAP3: six-transmembrane epithelial antigen of the prostate 3; DMT1: divalent metal transporter 1; FPN: ferroportin; PUFAs: polyunsaturated fatty acids; LOXs: lipoxygenases; ROS: reactive oxygen species; NCOA4: nuclear receptor coactivator 4; LIP: labile iron pool; GSSG: oxidized GSH; DHODH: dihydroorotate dehydrogenase; CoQH2: ubiquinol; GCH1: guanosine triphosphate (GTP) cyclohydrolase 1; BH2: dihydrobiopterin; BH4: tetrahydrobiopterin; HIF-2α: hypoxia inducible factor-2α; TfR1: Tf receptor 1.

#### 3.3.2 Ferroptosis in IVDD

Iron is essential for human physiology but contributes to IVDD when dysregulated. Excess iron promotes ferroptosis through lipid peroxidation and ROS accumulation, initiating cell death pathways. Pathogenic factors such as mechanical overload, inflammatory infiltration, and oxidative stress disrupt iron homeostasis, thereby triggering ferroptosis in NPCs, AFCs, and CEPCs, ultimately accelerating IVDD progression [61].

NPCs exhibit marked susceptibility to ferroptosis and oxidative stress, partly due to enhanced vulnerability to pro-methylation effects mediated by DNA methyltransferase 3B (DNMT3B) [62] or homocysteine [63], both of which promote iron overload. Experimental evidence shows that NPCs treated with oxidized low-density lipoprotein (Ox-LDL) display activation of the lectin-like Ox-LDL receptor 1 (LOX-1), which drives inflammatory signaling and lipid peroxidation. *In vivo*, lumbar instability mouse models demonstrate a strong positive correlation between IVDD severity and ferroptosis activity, evidenced by upregulation of LOX-1, prostaglandin G/H synthase 2, and NADPH oxidase 1, alongside decreased GPX4 and ferritin heavy chain (FTH) expression. Therapeutic interventions such as liproxstatin-1 (Lip-1) or LOX-1 silencing (shLOX-1) restore anti-ferroptotic markers, including GSH, thereby enhancing NPC viability and partially alleviating IVDD [64]. Similarly, needle-puncture rat models reveal that iron overload correlates significantly with Pfirrmann grade, accompanied by reduced FTH1 and GPX4 expression in degenerated IVDs. Treatment with the ferroptosis inhibitor ferrostatin-1 (Fer-1) effectively rescues NPC viability and mitigates ECM degradation [65].

Bioinformatic analyses suggest that ferroptosis-related genes, including *CREB3* and *MGST1*, play critical roles in AF degeneration-driven IVDD progression. *CREB3* expression is significantly downregulated in degenerated AF tissues and shows functional correlations with multiple immune cell subsets. This reduction in *CREB3* likely heightens AFCs susceptibility to ferroptosis by impairing ER stress repair mechanisms or disrupting anti-inflammatory signaling pathways, thereby accelerating IVDD pathogenesis [66]. In contrast, *MGST1* is markedly upregulated in degenerated AF tissues. Although its precise role in IVDD remains underexplored, *MGST1* may exert protective effects by maintaining the GSH/GSSG redox balance or suppressing lipid peroxidation chain reactions in AFCs, potentially delaying IVDD progression [67]. Moreover, under oxidative stress conditions, AFCs exhibit elevated lipid peroxidation, which accelerates ECM degradation and sustains IVDD advancement. Collectively, these findings provide mechanistic insight and highlight ferroptosis-related targets for therapeutic intervention in AF degeneration [68].

The healthy CEP supports nutrient diffusion into the IVD, whereas calcification and vascular invasion disrupt disc homeostasis. Iron overload in CEPCs induces mitochondrial damage and ECM degradation, ultimately promoting CEP mineralization and IVDD through ferroptosis [61]. The hypoxia inducible factor-2α (HIF-2α)/TfR1 pathway is central to this process: under pathological IVDD conditions, HIF-2α upregulates TfR1 expression, enhancing iron influx. Excess iron accumulation not only intensifies oxidative stress in CEPCs but also activates cyclic guanosine monophosphate-adenosine monophosphate (GMP-AMP) synthase/stimulator of interferon (IFN) genes (cGAS/STING)-mediated innate immune signaling via mitochondrial damage and mitochondrial DNA (mtDNA) release, thereby amplifying inflammatory cascades [69]. Conversely, CEPC-targeted engineered exosomes loaded with salvianolic acid A suppress intracellular HIF-2α/TfR1 expression following cellular uptake, reducing iron ion influx and ROS levels. In parallel, inhibition of the cGAS/STING pathway limits mtDNA replication and alleviates CEPC degeneration. These findings highlight the therapeutic potential of combining natural products with bioengineering strategies to counteract CEP ferroptosis and IVDD progression [70] (Fig. 4B).

#### 3.3.3 Natural products alleviate IVDD by targeting ferroptosis

Natural products demonstrate significant potential in combating ferroptosis and ameliorating IVDD through diverse regulatory mechanisms. These compounds can chelate iron ions to reduce intracellular free iron levels, thereby diminishing iron-mediated Fenton reactions and suppressing lipid peroxidation to mitigate oxidative damage to cellular membranes and organelles. Simultaneously, natural products facilitate maintaining mitochondrial function, enhance cellular energy metabolism, and restore redox homeostasis, collectively inhibiting ferroptosis progression and alleviating IVDD development through multiple molecular pathways [71].

The natural compound cynarin, derived from artichoke, activates the Nrf2/GPX4 signaling axis in NPCs, effectively neutralizing lipid peroxides and restoring redox homeostasis. By upregulating GPX4, cynarin reduces mitochondrial ROS accumulation and COL2 degradation while maintaining ECM integrity under oxidative stress, thereby protecting NP function [72]. The flavonoid fisetin, found in fruits and vegetables, not only alleviates oxidative stress in rat nucleus pulposus mesenchymal stem cells (NPMSCs) by modulating SIRT1 [73], but also enhances clearance of damaged mitochondria to inhibit ferroptosis in AFCs and preserve AF structural integrity [74]. Astaxanthin, a natural lipid-soluble red-orange carotenoid, mitigates CEP calcification by reducing oxidative stress, inflammation, and ferroptosis in CEPCs through enhanced activation of the Nrf2/heme oxygenase-1 (HO-1) pathway and suppression of nuclear factor kappaB (NF-κB) activity [75]. Similarly, tomatidine, a tomato-derived alkaloid, alleviates IVDD progression both *in vitro* and *in vivo* by activating the Nrf2/HO-1/GPX4 signaling axis while inhibiting ferroptosis in NPCs [76].

These natural compounds exert multifaceted effects on various stages of ferroptosis, including iron homeostasis regulation, mitochondrial integrity maintenance, lipid peroxidation inhibition, and ECM component protection. Their therapeutic efficacy has been validated in both *in vitro* and *in vivo* IVDD models, demonstrating the considerable potential of natural products as ferroptosis modulators for IVDD intervention (Table S3).

### 3.4 Pyroptosis

#### 3.4.1 Overview of pyroptosis

Pyroptosis was first identified in 1992 and later confirmed in Shigella-infected murine and human macrophages [77]. The term “pyroptosis” was introduced in 2001 to describe this inflammatory form of PCD. It is mediated by the gasdermin (GSDM) protein family, which shares approximately 45% sequence similarity. Each GSDM contains an autoinhibitory C-terminal domain and an effector N-terminal domain (GSDM-N); proteolytic cleavage releases the bioactive N-terminal fragment (NT). The defining hallmarks of pyroptosis include plasma membrane perforation and proinflammatory cytokine release, processes primarily driven by inflammatory caspase-mediated cleavage of gasdermin-D (GSDMD). The released GSDMD-NT oligomerizes to form 10−15 nm pores in the plasma membrane, ultimately leading to ionic gradient collapse, cell swelling, rupture, and lytic cell death [78].

GSDMD-dependent pyroptosis is initiated through two primary pathways. The canonical pathway begins with inflammasome assembly, where pattern recognition receptors such as NOD-like receptor NLR, family pyrin domain-containing 3 (NLRP3) or absent in melanoma 2 (AIM2) detect pathogen-associated molecular patterns (PAMPs) or damage-associated molecular patterns (DAMPs). The adaptor protein apoptosis-associated speck-like protein containing a caspase-activation and recruitment domain (CARD) (ASC) subsequently recruits and activates caspase-1, which cleaves GSDMD and processes pro-interleukin (IL-1β (IL-1β) into its active form. This sequence triggers a self-amplifying inflammatory cascade [79]. In contrast, the non-canonical pathway is mediated by caspase-4/5 in humans or caspase-11 in mice, which directly sense cytosolic lipopolysaccharide. This pathway bypasses inflammasome formation, leading to direct cleavage of GSDMD and pyroptosis induction. It is particularly relevant in Gram-negative bacterial infections but may also be activated by other intracellular danger signals [78,80].

Beyond GSDMD, other GSDM family members (GSDM A/B/C/E) can also mediate pyroptosis through distinct activation mechanisms. For example, GSDMA is activated by the streptococcal pyrogenic exotoxin B from group A *Streptococcus* (GAS), GSDMB by granzyme A released from cytotoxic T lymphocytes and natural killer (NK) cells [81], and GSDMC and GSDME by caspase-8 and caspase-3 cleavage, respectively. Despite their different upstream activators, all generate membrane pores to execute pyroptosis. In addition, recent studies have revealed GSDM-independent pyroptotic pathways. One example involves phosphorylated mixed lineage kinase domain-like protein (MLKL), which disrupts plasma membrane integrity while simultaneously promoting NLRP3 inflammasome assembly and caspase-8 activation, thereby facilitating pore formation. This mechanism may represent an evolutionary redundancy, ensuring the induction of inflammatory cell death during viral infection or immune responses to preserve host defense [82] (Fig. 5A).

**Fig. 5:**
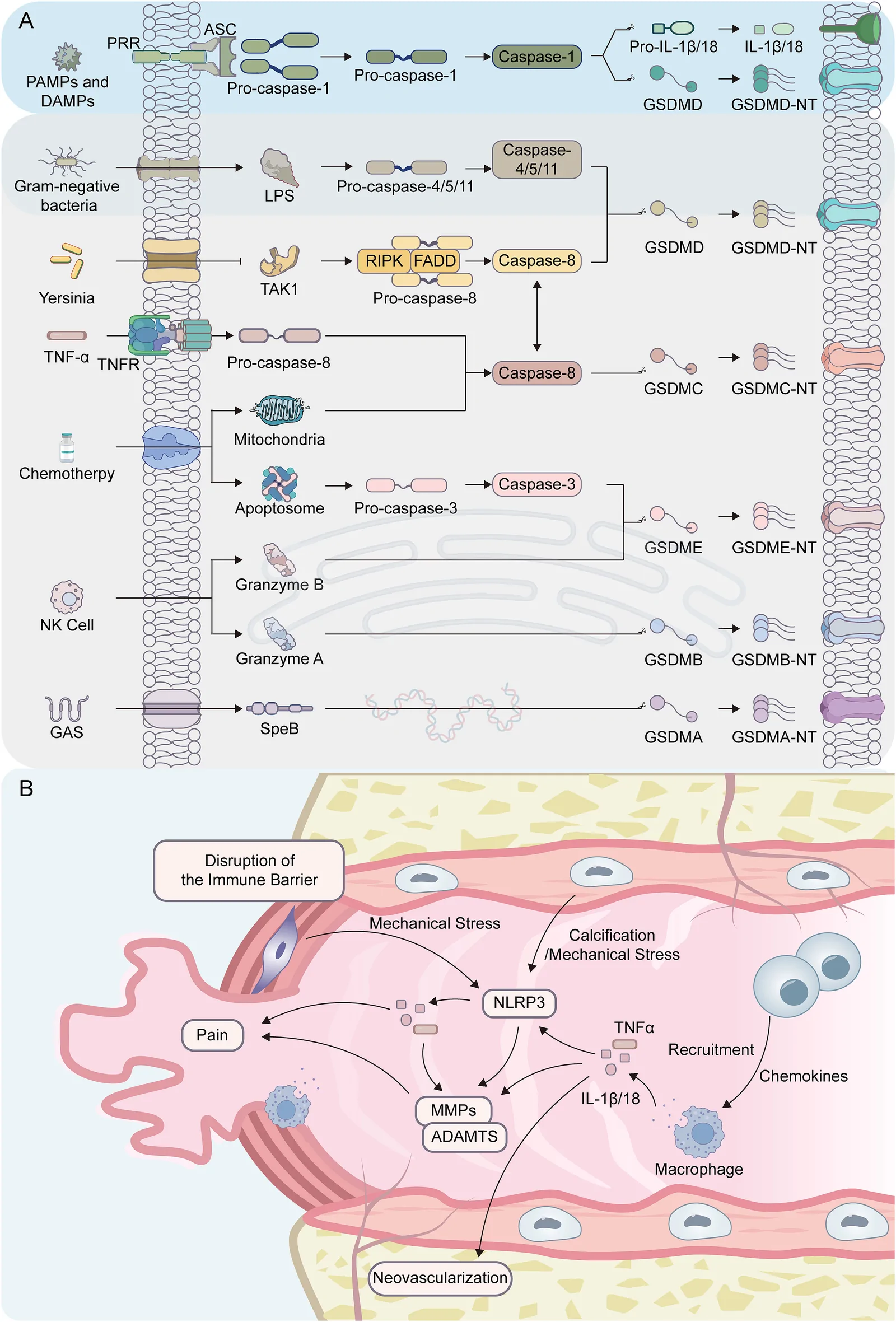
Mechanisms of pyroptosis in intervertebral disc (IVD) degeneration (IVDD). (A) Pyroptosis is primarily mediated by canonical caspase-1-dependent and non-canonical (caspase-4/5/11-dependent) pathways that cleave gasdermin (GSDM)-D (GSDMD) to form membrane pores for interleukin (IL)-1β/IL-18 release, with alternative pathways directly activating other GSDM members. (B) In IVDD, nucleus pulposus (NP) barrier disruption initiates pyroptosis in NP cells (NPCs), establishing a pro-inflammatory cycle that recruits macrophages, accelerates extracellular matrix (ECM) degradation, and propagates disease through mechanochemical and immunological dysregulation. PAMPs: pathogen-associated molecular patterns; DAMPs: damage-associated molecular patterns; PRR: pattern recognition receptors; ASC: apoptosis-associated speck-like protein containing a caspase activation and recruitment domain (CARD); GSDMD-NT: GSDMD-N-terminal; LPS: lipopolysaccharide; TAK1: transforming growth factor-β (TGF-β)-activated kinase 1; RIPK: receptor-interacting protein (RIP) kinase; FADD: Fas-associated death domain; TNF-α: tumor necrosis factor-α; TNFR: TNF receptors; GSDMC: gasdermin C; GSDME: gasdermin E; NK cell: natural killer cell;GSDMB: gasdermin B; GAS: group A *Streptococcus*; SpeB: streptococcal pyrogenic exotoxin B; GSDMA: gasdermin A; NLRP3: NOD-like receptor NLR, family pyrin domain-containing 3; MMPs: matrix metalloproteinases; ADAMTS: a disintegrin and metalloproteinase with thrombospondin motifs.

#### 3.4.2 Pyroptosis in IVDD

Current evidence indicates that pyroptosis in NPCs plays a well-established role in IVDD. Degenerated IVD tissues consistently exhibit significant upregulation of pyroptosis-related molecules, including NLRP3, caspase-1, and IL-1β, at both messenger RNA (mRNA) and protein levels, with their expression showing a positive correlation with IVDD severity [83].

The NLRP3 inflammasome in NPCs is activated by various endogenous DAMPs, driving the release of pyroptosis-associated inflammatory cytokines such as IL-1. This cytokine not only functions as a structural remodeling mediator by elevating a disintegrin and metalloproteinase with thrombospondin motifs (ADAMTS) levels in the NP, but also stimulates NPCs to secrete chemokines that recruit immune cells, thereby facilitating macrophage and neutrophil infiltration [84]. Moreover, even in the absence of stimuli strong enough to induce overt structural NP damage, Piezo1-mediated pyroptosis can still occur in NPCs [85]. Collectively, these findings suggest that pyroptosis may represent an early and critical event in IVDD, acting as a regulatory hub that links IVD homeostasis with matrix synthesis and metabolic balance.

AFCs play a pivotal role in maintaining the structural integrity and stability of IVDs, and their damage, similar to that of NPCs, acts as a key trigger for cytokine and chemokine accumulation within the disc [84]. Although research on AFC pyroptosis is still limited, available evidence strongly supports its association with IVDD progression. Both H_2_O_2_ and ubiquitin-specific protease 14 (USP14) interventions can induce AFC pyroptosis, the former through pro-oxidative stress and the latter via NLRP3 deubiquitination [86]. In addition to these chemical inducers, high-intensity mechanical stress or lumbar instability can also provoke pyroptosis in AFCs [87]. Collectively, IVD degeneration is substantially driven by elevated inflammatory factors and NLRP3 inflammasome activation in AFCs, which induce pyroptosis-mediated morphological changes and functional impairment, ultimately leading to AF microdamage, reduced tensile strength, and IVDD pathogenesis.

CEPCs are essential for nutrient exchange and mechanical transduction within the IVD. In IVDD patients, degenerated CEPs frequently display pathological changes such as structural tearing and calcification, which may be linked to pyroptosis triggered by lipid metabolism dysregulation and impaired disc blood flow [88]. In these tissues, NLRP3 inflammasome activation markedly increases the expression of pyroptosis-associated proteins, including caspase-1 and IL-1β [89]. Similarly, ECM degradation and ectopic ossification observed in CEPs of IVDD mouse models have also been associated with pyroptotic activity [90]. Collectively, these findings indicate that CEPC pyroptosis compromises endplate structural and functional integrity by promoting CEP fibrosis, thereby impairing blood supply, nutrient transport, and mechanical stability. This creates a chronically unfavorable microenvironment that accelerates IVD degeneration (Fig. 5B).

#### 3.4.3 Natural products alleviate IVDD by targeting pyroptosis

Emerging as a novel therapeutic strategy for IVDD, pyroptosis-targeted modulation has garnered increasing research attention, with several natural products demonstrating efficacy in alleviating IVDD progression through regulation of pyroptosis-related pathways. Icariin exhibits significant protective effects against IL-1β-induced inflammatory responses in NPCs, potentially attenuating IVDD progression via suppression of NLRP3-mediated pyroptosis [91]. Morin, a flavonol isolated as a yellow pigment from plants belonging to the Moraceae family, ameliorates IVDD by inhibiting the thioredoxin-interacting protein (TXNIP)/NLRP3/caspase-1/IL-1β signaling axis and subsequently reducing pyroptosis in NPCs [92]. Notoginsenoside R1, a principal bioactive compound derived from *Panax notoginseng*, demonstrates therapeutic efficacy by suppressing the NF-κB/NLRP3 pathway in NPCs. This inhibition leads to attenuated pyroptosis intensity, resulting in improved NPC functionality and significant alleviation of both mechanical allodynia and thermal hyperalgesia in puncture-induced IVDD rat models [93]. These findings substantiate the therapeutic potential of natural products in modulating pyroptosis through distinct molecular pathways to mitigate IVDD progression, thereby offering promising prospects for developing safer and more effective treatment modalities for IVDD patients (Table S4).

### 3.5 Necroptosis

#### 3.5.1 Overview of necroptosis

Necroptosis is a regulated form of PCD that can be pharmacologically modulated. The core molecular framework of necroptosis was further elucidated in 2012, when RIPK3 and MLKL were identified as essential effector molecules [94].

Necroptosis is characterized by cell swelling, plasma membrane rupture, mitochondrial dysfunction, and the release of DAMPs, in sharp contrast to the preserved membrane integrity observed in apoptosis [95]. The process is mediated by a tightly regulated RIPK1/RIPK3/MLKL signaling cascade and can be initiated by diverse stimuli, including TNFR activation, Toll-like receptor (TLR) signaling, or pathogen-associated factors. In the TNFR1 pathway, receptor engagement forms complex II, which consists of FADD and caspase-8. When caspase-8 is active, RIPK1 is cleaved, driving apoptosis. However, inhibition of caspase activity promotes RIPK1-RIPK3 oligomerization through their RIP homotypic interaction motif (RHIM) domains, leading to necrosome formation [96]. Similarly, TLR3/4 signaling via the TRIFosome or activation of Z-DNA-binding protein 1 (ZBP1) through RHIM-dependent interactions recruits and activates RIPK3, thereby initiating necrosome assembly and triggering necroptosis [97].

MLKL activation represents the terminal step of necroptosis. RIPK3 phosphorylates the pseudokinase domain of MLKL, inducing conformational changes that unlock its membrane-disruptive activity. Current research is centered on elucidating how activated MLKL interacts with the plasma membrane. Several models have been proposed, including the formation of nanopores, cation-selective channels, or amyloid-like fibrils [98]. Nonetheless, the precise molecular mechanisms underlying MLKL-mediated membrane disruption remain incompletely defined (Fig. 6A).

**Fig. 6:**
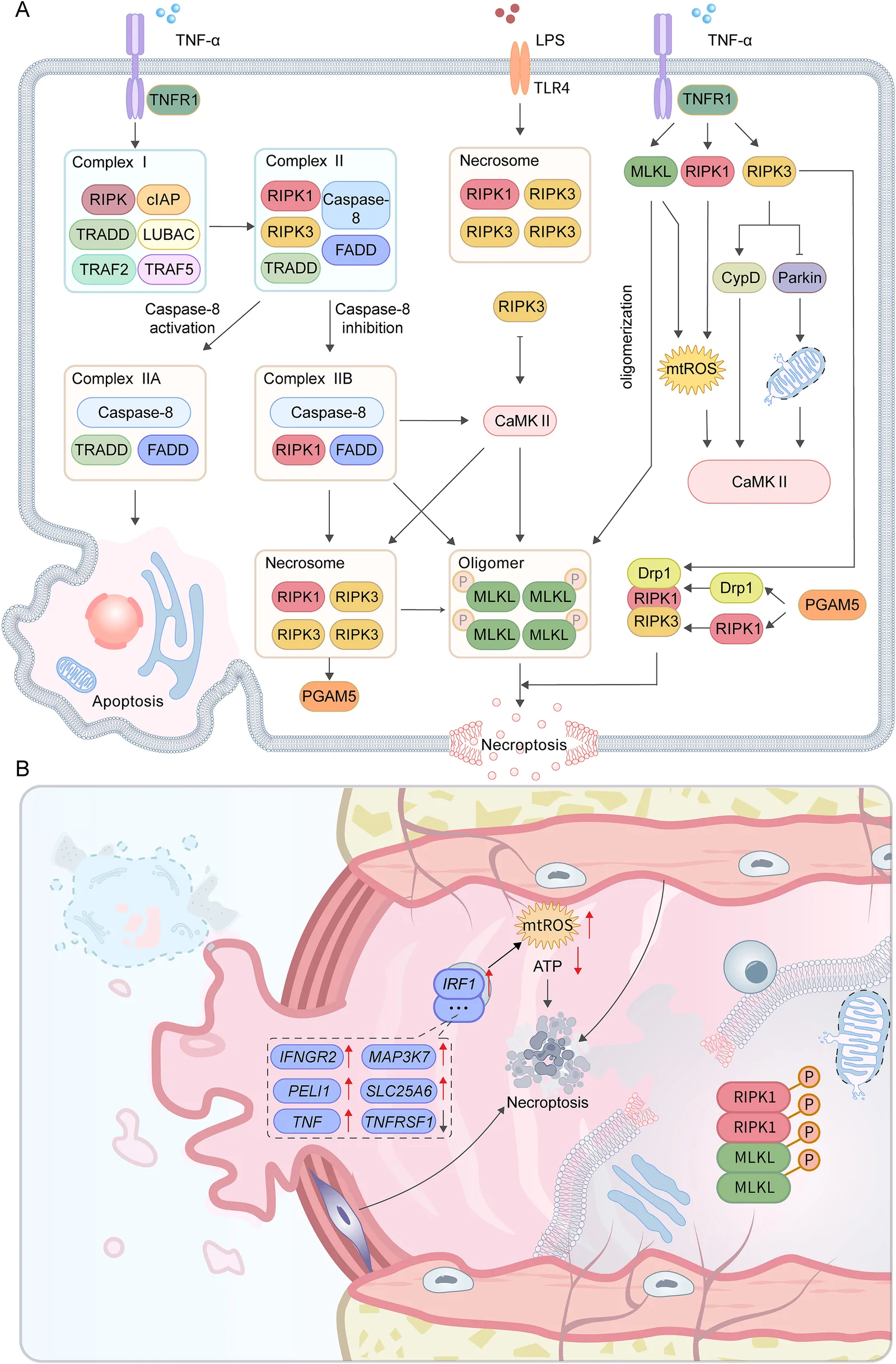
Mechanisms of necroptosis in intervertebral disc (IVD) degeneration (IVDD). (A) Necroptosis is triggered by tumor necrosis factor (TNF) signaling through TNF receptor 1 (TNFR1), leading to receptor-interacting protein (RIP) kinase (RIPK) 1/RIPK3-mediated phosphorylation of mixed lineage kinase domain-like protein (MLKL) and plasma membrane disruption. (B) In IVDD, necroptosis-apoptosis crosstalk is regulated by caspase-8 activity, with TNF/TNFR pathway activation prominent in advanced stages, while calcium/calmodulin-dependent protein kinase II (CaMKII) emerges as a RIPK3-regulated target influencing mitochondrial dysfunction in musculoskeletal contexts. LPS: lipopolysaccharide; TLR4: Toll-like receptor 4; cIAP: cellular inhibitor of apoptosis protein; TRADD: TNFR1-associated death domain protein; LUBAC: linear ubiquitin chain assembly complex; TRAF2: TNFR-related factor 2; FADD: Fas-associated death domain; CypD: cyclophilin D; mtROS: mitochondrial reactive oxygen species (ROS); PGAM5: phosphoglycerate mutase family member 5; Drp1: dynamin-related protein 1;ATP: adenosine triphosphate.

#### 3.5.2 Necroptosis in IVDD

Recent advances in bioinformatics and machine learning have greatly expanded our understanding of the molecular mechanisms of necroptosis in IVDD. Systematic bioinformatic analyses have identified necroptosis as one of the four predominant PCD pathways implicated in IVDD, with the Bcl-2 family protein BH3-interacting domain death agonist (BID) emerging as a potential upstream regulatory target [99]. Moreover, *IRF1*, *PDCD6*, and *SUBE2K* have been recognized as core regulatory genes [100,101]. Single-cell transcriptomic sequencing has further uncovered cell-subpopulation-specific heterogeneity in necroptosis-related gene expression within IVD tissues. Notably, *NT5E* and *TMEM158* were identified as putative central regulators of IVDD-associated necroptosis, showing preferential expression in fibrochondrocytes and inflammatory chondrocytes rather than the traditionally studied NPCs. These findings establish a novel molecular stratification framework that could enable more precise, cell type-specific therapeutic targeting of necroptosis in IVDD [102].

Necroptosis of NPCs markedly accelerates IVDD progression. Mechanical stress, a well-established inducer of IVDD, has been shown to drive necroptosis in rat NPCs: with increasing mechanical loading, these cells progressively exhibit necroptosis-like morphological changes accompanied by elevated expression and phosphorylation of RIPK1, RIPK3, and MLKL over time. Pharmacological inhibition using the RIPK1 inhibitor Nec-1, the RIPK3 inhibitor GSK'872, or the MLKL inhibitor NSA significantly attenuates necroptosis and partially restores cellular viability [103]. Oxidative stress likewise acts as a potent trigger, as H_2_O_2_ treatment upregulates RIP1 and RIP3 expression while activating the PARP-AIF pathway to induce NPC necroptosis. Notably, these changes are effectively suppressed by Nec-1 treatment [104].

Current research on necroptosis in AFCs and CEPCs during IVDD progression remains limited. Existing evidence indicates that AFCs exhibit elevated expression of RIPK3, MLKL, and p-MLKL, suggesting a predisposition to necroptosis in IVDD pathogenesis [105]. The scarcity of studies on these cell types largely arises from their structural and functional complexities: AFCs are difficult to model due to their intricate lamellar organization and mechanically dynamic microenvironment, whereas CEPC research has predominantly focused on nutrient metabolism rather than PCDs. As a result, the necroptotic features of both cell populations remain systematically uncharacterized. These knowledge gaps not only hinder a comprehensive understanding of necroptosis in IVDD but also highlight important avenues for future exploration. Promising directions may include the development of biomimetic mechanical loading systems to replicate the physiological stress environment of CEPs, or the use of biomaterial scaffolds to mimic the layered architecture of the AF, tools that could enable deeper investigation of necroptosis in these underexplored IVD compartments [106] (Fig. 6B).

#### 3.5.3 Natural products alleviate IVDD by targeting necroptosis

Currently, there exists limited reliable evidence regarding the modulation of necroptosis in IVD by natural products for alleviating IVDD. To date, limonin remains the only natural product conclusively demonstrated to suppress necroptosis in IVDD. Mechanistically, limonin exerts its therapeutic effects by inhibiting the RIP1/RIP3/MLKL signaling pathway, thereby reducing necroptosis in NPCs. This inhibition subsequently attenuates inflammatory responses and ECM degradation, ultimately maintaining disc structural stability and retarding IVDD progression [107]. Overall, the current evidence supporting necroptosis-targeted therapies for IVDD remains insufficient due to the challenges in precisely determining the specific promotive effects of necroptosis on IVDD progression and elucidating its interactions with other well-characterized forms of PCD beyond apoptosis that have been clearly implicated in IVDD pathogenesis. Nevertheless, several natural products have demonstrated necroptosis-modulating capabilities in other disease contexts: *Ampelopsis grossedentata* [108] exerts hepatoprotective effects against alcoholic liver disease by specifically inhibiting the TLR4/NF-κB signaling axis and blocking MLKL phosphorylation-mediated hepatocyte injury; cardamonin [109], functioning as a selective small-molecule inhibitor of RIPK1/3 kinase activity, effectively prevents necrosome formation in intestinal epithelial cells and alleviates dextran sulfate sodium (DSS)-induced colitis by suppressing inflammatory cytokine production and epithelial barrier disruption; celastrol [110] mitigates necroptosis in acute pancreatitis models through multi-target inhibition of the RIPK1/RIPK3/MLKL pathway, suppression of ROS-dependent MLKL oligomerization, and maintenance of mitochondrial integrity. These established examples may provide valuable conceptual frameworks for necroptosis-related research in IVDD, potentially offering novel directions for developing necroptosis-targeted therapeutic strategies against IVDD.

### 3.6 Natural products regulate PANoptosis for IVDD

#### 3.6.1 Overview of PANoptosis

PANoptosis, first described in 2016 and systematically defined in 2019, derives its acronym from pyroptosis, apoptosis, and necroptosis (“P”, “A”, and “N”, respectively). It represents a distinct form of inflammatory PCD that integrates features of all three pathways and cannot be fully explained by any single mechanism [111].

Among PCDs, pyroptosis, apoptosis, and necroptosis are the most mechanistically well characterized. Initially thought to function independently, these pathways are now recognized to exhibit significant interconnections. Their crosstalk extends beyond previous assumptions: although each follows a distinct signaling route, they are often co-activated in pathological conditions, and cellular states can redirect similar upstream signals toward divergent fates. A representative example is influenza A virus (IAV)-induced PANoptosis [112]. During IAV infection, the innate immune sensor ZBP1 initiates assembly of the ZBP1-PANoptosome, which simultaneously activates caspase-1, caspase-3, caspase-8, and MLKL phosphorylation, ultimately driving lytic inflammatory death. Importantly, inhibition of any single PCD pathway does not prevent this outcome, underscoring the cooperative interplay and sophisticated regulatory network that form the conceptual foundation of PANoptosis [113].

Molecularly, PANoptosis is orchestrated through interactions among core components of multiple PCD pathways. At its center lies the PANoptosome, a dynamic signaling hub assembled via homotypic and heterotypic protein domain interactions. This supramolecular complex integrates upstream signals to activate downstream effectors and synchronize the execution of pyroptosis, apoptosis, and necroptosis [114]. The PANoptosome is composed of signaling adaptors and effectors that regulate cell death in a context-dependent manner. Upstream sensors, including ZBP1, AIM2, RIPK1, and NLRP12, detect pathogenic or stress signals to initiate the formation of specialized PANoptosomes (e.g., the ZBP1-PANoptosome), each featuring distinct combinations of sensors and regulators tailored to specific stimuli [115,116]. The downstream effector network incorporates caspase-6 and caspase-8 (apoptosis), NLRP3 (pyroptosis), and RIPK3 (necroptosis), thereby conferring broad regulatory capacity over multiple cell death modalities [117] (Fig. 7).

**Fig. 7:**
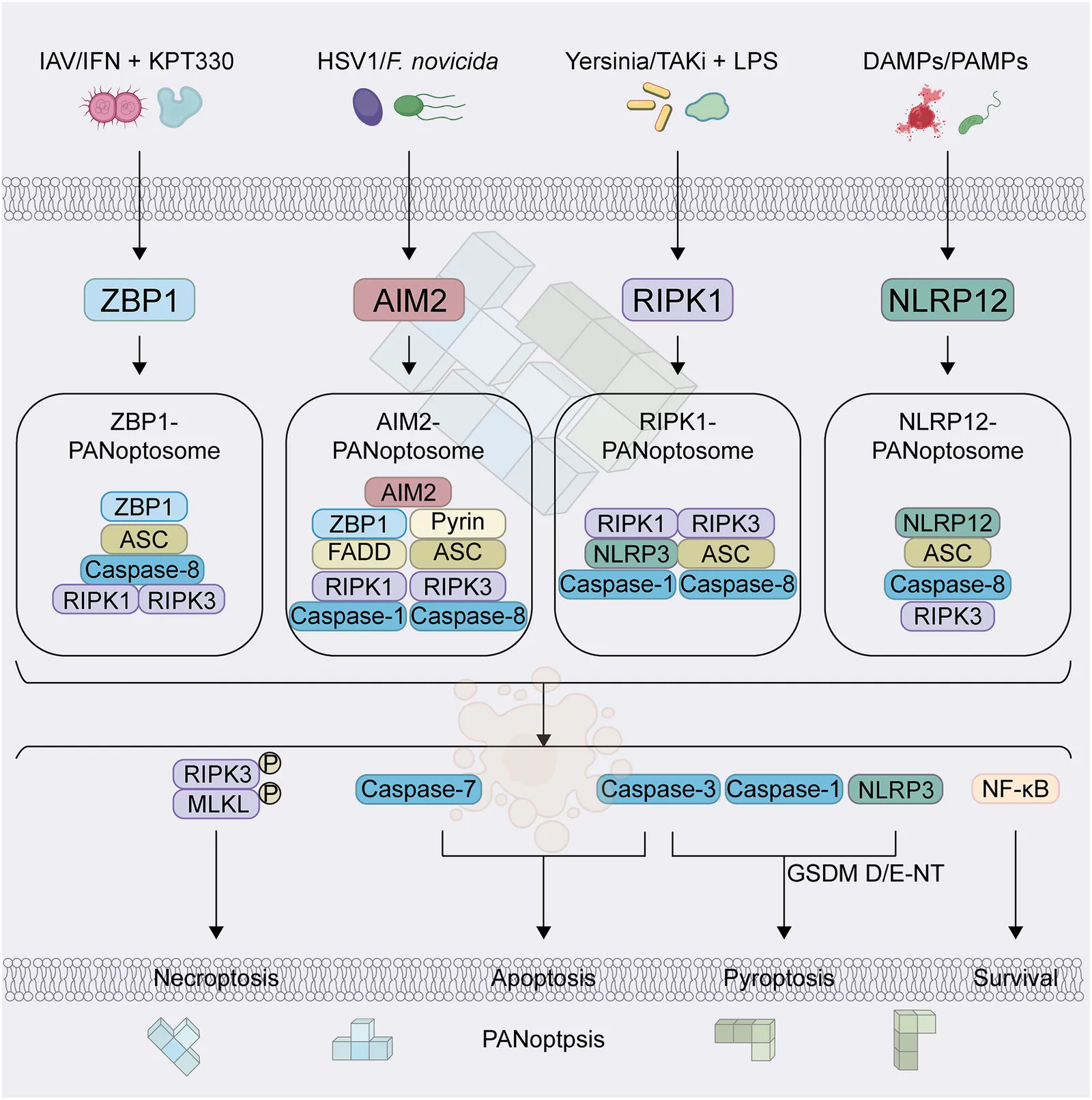
Mechanisms of PANoptosis. PANoptosis represents a systemic cell death network orchestrated by the coordinated interplay of apoptosis, pyroptosis, and necroptosis, mechanistically characterized by the assembly of PANoptosomes. To date, four sensor-defined PANoptosome subtypes have been identified: Z-DNA-binding protein 1 (ZBP1)-, absent in melanoma 2 (AIM2)-, receptor-interacting protein (RIP) kinase 1 (RIPK1)-, and NLR family pyrin domain-containing 12 (NLRP12)-driven complexes. These supramolecular structures mediate both homotypic and heterotypic intermolecular interactions, serving dual roles as platforms for proteolytic processing of cell death executors and as signaling hubs for death signal amplification. In intervertebral disc (IVD) degeneration (IVDD), the AIM2-PANoptosome is hypothesized to dominate the PANoptotic cascade. IAV: influenza A virus; IFN: interferon; HSV1: herpes simplex virus 1; *F. novicida*: *Francisella novicida*; TAKi: transforming growth factor-β (TGF-β)-activated kinase inhibitor; LPS: lipopolysaccharide; DAMPs: damage-associated molecular patterns; PAMPs: pathogen-associated molecular patterns; ASC: apoptosis-associated speck-like protein containing a caspase-activation and recruitment domain (CARD); FADD: Fas-associated death domain; MLKL: mixed lineage kinase domain-like protein; NF-κB: nuclear factor kappaB; GSDM: gasdermin; GSDM D/E-NT: gasdermin D/E-N-terminal.

#### 3.6.2 PANoptosis in IVDD

As a recently identified form of PCD, research on PANoptosis in IVDD is still in its early stages. Nevertheless, regulatory signatures of PANoptosis have been characterized in osteoarthritis (OA), offering valuable insights into potential mechanisms for its targeted modulation. OA pathogenesis is closely associated with tissue senescence and chronic inflammation, which create conditions conducive to PANoptosis in OA-relevant cells, thereby driving disease progression [118]. In this context, inflammatory mediators commonly initiate necroptosis via TNF-R1 activation, and the resulting release of DAMPs subsequently engages core pyroptosis effectors, upregulates MMP family proteins, and promotes ECM degradation, culminating in tissue damage [119]. Similar pathogenic mechanisms are present during IVDD progression. Cellular senescence is a primary risk factor, and chronic inflammation persists throughout disease development. The characteristic overexpression of MMP family proteins and subsequent ECM breakdown directly impair IVD structure and function. These parallels provide important reference points for investigating the potential role and regulatory mechanisms of PANoptosis in IVDD [120].

Recent advancements are gradually elucidating the regulatory landscape of PANoptosis in IVDD, with most insights derived from studies on NPCs. For example, a bioinformatic analysis integrating five Gene Expression Omnibus (GEO) datasets identified *GSDME* and *BID* as key genes regulating PCD in IVDD, both of which also function as critical molecular nodes within the PANoptosis network [99]. Additionally, Zhou et al. [121] provided experimental validation for PANoptosis-targeted interventional strategies. Through combined bioinformatic and experimental approaches, they identified *APAF1,* Mediterranean fever gene, *NLRP3*, *TNF*, *GSDMD*, *AIM2*, and IFN regulatory factor 1 (*IRF-1*) as central regulators of PANoptosis-mediated IVDD pathogenesis. These findings underscore the impact of PANoptosis on IVDD, suggesting that a predominantly pyroptosis-driven form of damage in NPCs may play a pivotal role during disease progression. The study further identified andrographolide as a natural product capable of directly modulating PANoptosis in NPCs, mitigating IVD damage and restoring intervertebral space height by regulating the expression of these PANoptosis-related genes, thereby alleviating IVDD progression.

#### 3.6.3 Natural products alleviate IVDD by targeting PANoptosis

Currently, few natural products have been experimentally confirmed to preserve IVD cellular homeostasis through PANoptosis regulation. Beyond the aforementioned andrographolide, kongensin A has been identified to attenuate IVDD by suppressing TAK1-mediated PANoptosis in NPCs. Mechanistically, these compounds exhibit distinct intervention strategies: kongensin A primarily inhibits upstream signal transduction by targeting TAK1 kinase activity, thereby disrupting the entire signaling cascade, whereas andrographolide more likely directly impedes the oligomerization of key protein complexes and PANoptosome assembly [122], representing two complementary therapeutic approaches for PANoptosis modulation in IVDD.

Despite limited current research, systematically characterizing how natural products modulate PANoptosis to alleviate IVDD remains challenging. However, existing findings still provide valuable insights to advance clinical IVDD management. Various traditional Chinese medicine formulations have demonstrated therapeutic effects against PANoptosis-induced conditions such as cardiomyopathy [123], by restricting PANoptosome assembly, broadly suppressing inflammatory cascades, and correcting aberrant PCD states. Natural products exhibit more specific mechanisms: *Achyranthes aspera* extract downregulates genes and signaling pathways related to oxidative stress, ER stress, inflammation, and PANoptosis [124], while scutellarin inhibits mitochondrial damage, reduces mtROS production, and blocks PANoptosome formation in bone marrow-derived macrophages, thereby improving macrophage polarization and mitigating multi-organ inflammatory responses [125]. Collectively, natural products exhibit inherent polypharmacological properties that can simultaneously target the multi-signal activation features of PANoptosis. Their capacity for multi-target modulation across PANoptosis activation pathways enables comprehensive inhibition of diverse cell death modalities in IVDD, offering promising potential for integrated, systematic therapeutic regulation.

The precise role of PANoptosis in IVDD progression and microenvironmental regulation remains poorly understood. Critical knowledge gaps persist regarding the biochemical characteristics of key molecular targets in PANoptosis, their activation mechanisms, the differential susceptibility of cells in IVD, and the dynamic transition nodes between “P", “A", and “N" components during IVDD advancement [126]. Therefore, targeting PANoptosis for IVDD intervention still faces significant challenges. However, as research progresses, these critical questions are expected to be gradually resolved, potentially paving the way for novel therapeutic strategies against IVDD.

## 4 Mechanisms and therapeutic strategies

### 4.1 Crosstalk and fate decisions in the PCD network

Different PCDs exhibit variable sensitivity to specific stimuli, and even under identical conditions, distinct cell types or cellular states can respond differently. For example, during IVDD progression, the predominant apoptotic pathway correlates with disease stage: ER stress-mediated apoptosis predominates in the early stages, whereas death receptor- and mitochondrial-mediated pathways become more prominent in intermediate to advanced stages, as evidenced in clinical IVD samples [127]. These observations illustrate the dynamic evolution of PCD patterns during disease progression and help explain why trauma-induced IVDD often triggers premature activation of death receptor pathways, because acute trauma and inflammation may accelerate death receptor binding and activation [128]. Such insights are crucial for developing stage-specific therapeutic strategies, as identifying the dominant PCD types and their temporal dynamics may enable personalized interventions to counteract the deleterious effects of PCD dysregulation during IVDD.

In contrast to apoptosis, which is readily triggered by inflammatory factors, autophagy in AFCs exhibits distinct activation patterns. Inflammatory stimuli alone rarely elicit significant autophagic responses, whereas serum starvation effectively induces autophagy, indicating that this process primarily responds to endogenous cues, such as nutrient deprivation, rather than directly countering external threats. Functionally, autophagy acts both as a “buffer”, mitigating cellular stress accumulation, and as a “lubricant”, enhancing overall cellular defense mechanisms. The timing of autophagic activation is critical in determining its biological impact: early-stage autophagy provides essential metabolic support and preserves intracellular homeostasis under stress conditions [45], whereas excessive or late-stage autophagy can exacerbate IVD degeneration by promoting cellular self-digestion [129].

This ubiquitous yet “paradoxical” phenomenon, exhibiting both protective and detrimental effects, renders the precise role of autophagy in IVDD pathogenesis ambiguous, suggesting a dual functionality during disease progression [130]. Under mild but prolonged stress conditions, such as nutrient deprivation or chronic inflammation, autophagy functions as a conserved adaptive mechanism, mitigating mitochondrial dysfunction and upregulating COL2 and aggrecan expression to preserve ECM homeostasis. Conversely, sustained high autophagic flux under persistent stress can lead to excessive cellular degradation, ultimately causing cellular damage. Given its intrinsic roles in energy recycling and metabolic regulation, autophagy is likely critical for maintaining the metabolic stability of IVD cells within their nutrient-deprived microenvironment. Therefore, moderate autophagic activity appears essential for sustaining homeostasis within the relatively isolated IVD ecosystem [51].

Current evidence suggests that natural products primarily alleviate IVDD by moderately enhancing autophagic flux. Strong autophagic activation is typically induced by intense pathological stressors, such as severe oxidative damage, acute inflammatory signaling, or extreme nutrient deprivation, conditions under which most natural products exhibit limited efficacy. In these scenarios, the primary pathological drivers demand prioritized intervention, and the stress intensity often exceeds the physiological regulatory capacity of natural compounds. This indicates that the therapeutic benefits of natural products are likely most pronounced during the early to middle stages of IVDD, when cellular stress remains relatively moderate. Further elucidation of the molecular targets and spatiotemporal dynamics underlying natural product-mediated autophagy modulation could facilitate the development of more precise, stage-specific, and temporally controlled autophagic therapies.

The caspase-8/RIPK1 axis serves as a critical molecular switch governing cell fate decisions between apoptosis and necroptosis. Activation of caspase-8 promotes apoptotic signaling, whereas its inhibition facilitates RIPK1/3-dependent MLKL phosphorylation, redirecting PCD toward necroptosis and highlighting the intricate interplay between these pathways [117]. Notably, MLKL pore-mediated potassium efflux during necroptosis can activate the NLRP3 inflammasome [131], and more directly, MLKL participates in TLR3-mediated NLRP3 activation, as MLKL deficiency impairs ASC oligomerization and subsequent pyroptosis [132]. Similarly, ferroptosis engages in cross-talk with pyroptosis: ACSL4-dependent ferroptosis generates 12-hydroxyeicosatetraenoic acid via arachidonate 12-LOX, which upregulates NLRP3, ASC, and GSDMD expressions [133]. These adaptive activation mechanisms and pleiotropic effects illustrate the remarkable plasticity of PCDs in responding to environmental cues, collectively forming a sophisticated regulatory network of interconnected cell death modalities.

### 4.2 Beyond binary inhibition

Given the critical roles and broad regulatory influence of key targets such as caspase-8, one might ask whether selective inhibition of caspase-8 could suppress PCDs and enhance cellular viability. The answer is no. Complete knockout of caspase-8 or its adaptor protein FADD results in embryonic lethality around10.5 days in mice, with viability restored only upon concurrent deletion of RIPK3 or MLKL [134]. This underscores the indispensable role of caspase-8 in embryonic development within the PCD network, where even partial inhibition, as observed in caspase-8^FGLG/FGLG^ mice, renders animals hypersensitive to endotoxin-induced lethality. Similarly, apoptosis-deficient mice either die during embryogenesis or shortly after birth, and the few survivors display multiple pathological abnormalities [135].

The underlying rationale stems from the principle that PCDs maintain homeostasis through balanced regulation, rather than through unidirectional promotion or inhibition of individual pathways. Their core biological significance lies in synergistic and complementary interactions that preserve tissue integrity. PCDs do not function in isolation; instead, they engage in dynamic molecular crosstalk, forming an integrated “death network” that flexibly adapts to microenvironmental changes. This network architecture serves dual protective functions: it prevents tissue damage caused by overactivation of a single PCD pathway and provides compensatory mechanisms when specific PCDs fail, thereby averting systemic collapse. Consequently, therapeutic strategies targeting the PCD network must move beyond binary “all-or-nothing” approaches aimed at individual pathways. Instead, emphasis should be placed on identifying pivotal nodal points that act as “gatekeepers” across multiple pathways, enabling spatiotemporal recalibration of PCD equilibrium. Such a paradigm involves multidimensional disease interception by simultaneously preventing pathological tissue loss from excessive cell death and avoiding dysfunction from inadequate cellular clearance, ultimately restoring physiological homeostasis through precision modulation of the death network.

### 4.3 Challenges in the clinical translation

Multiple PCDs play critical roles in IVDD progression, and natural products targeting these pathways exhibit promising therapeutic potential. However, clinical translation remains challenging due to the complex dynamics of PCDs and the current lack of reliable methods for quantitatively monitoring these processes. This limitation hinders the development of PCD-based diagnostic and prognostic tools, thereby constraining the implementation of targeted interventions in clinical practice.

#### 4.3.1 Challenges in mechanistic elucidation

The regulatory effects of natural products on IVDD are multifaceted, encompassing oxidative stress modulation, suppression of inflammatory responses, regulation of signaling pathways, and control of gene expression. While these broad-spectrum effects confer significant therapeutic potential, they also complicate mechanistic elucidation [136]. Although some key pathways and targets of specific natural products have been identified, the interplay among these pathways and the underlying regulatory networks remain incompletely understood. Moreover, the existence of additional pivotal molecules and the mechanisms by which they may synergistically interact with known targets warrant further in-depth investigation.

#### 4.3.2 Challenges in standardization

From the perspective of natural product properties and regulatory characteristics, biologically derived therapeutic compounds present unique challenges in standardization and quality control. As these substances are extracted from plants or animals, their active ingredient content and pharmacological properties are influenced by factors such as geographical origin, harvest season, and extraction methods. Consequently, compositional variations between production batches are inevitable, making the establishment of highly consistent quality standards a complex but essential task.

Taking mangiferin as an example, this bioactive compound, known for its antidiabetic and antioxidant activities, is present in plant families such as Anacardiaceae, Gentianaceae, and Iridaceae, but accumulates most abundantly in mango trees (*Mangifera indica* L.). Research indicates substantial variation in mangiferin distribution among mango tissues: approximately 4 mg/kg in pulp, 42 mg/kg in seed kernels, and 1690 mg/kg in peels [137]. Moreover, extraction efficiency is highly dependent on methodology, with Soxhlet extraction, sonication, and microwave-assisted techniques demonstrating distinct performance characteristics [138]. These technical considerations highlight that translating promising natural products like mangiferin from basic research to clinical application necessitates the development of efficient, standardized extraction processes.

#### 4.3.3 Challenges in safety assessment

From a clinical translation perspective, the successful application of natural products requires the establishment of a comprehensive safety evaluation system. Although natural products constitute a valuable resource for drug discovery, their complex chemical composition and diverse biological origins necessitate rigorous and scientifically robust assessment approaches. Notably, certain traditional Chinese medicines have been associated with adverse effects, underscoring the importance of toxicological evaluation as a critical consideration in the future clinical development of natural products.

Traditional approaches to natural product safety assessment have primarily relied on historical documentation and reports of adverse reactions. Moving forward, safety evaluations must integrate modern toxicological research to establish precise, quantitative indicators and standards that align with contemporary drug development and regulatory requirements. Particular emphasis should be placed on assessing the safety of long-term use, necessitating the implementation of continuous monitoring and evaluation mechanisms to ensure sustained efficacy and patient protection [139].

Consequently, the development of novel evaluation models and molecular-level detection frameworks is imperative. Quantifying relevant biomarkers and elucidating dose-response-toxicity relationships will advance the scientific development and rational utilization of natural products [140]. Moreover, comprehensive investigation of their pharmacokinetic properties, including absorption, distribution, metabolism, and excretion, is essential for designing optimal dosing regimens that achieve a balance between efficacy and safety [141].

#### 4.3.4 Challenges in modeling

The limitations of current IVDD models pose significant challenges for translating natural product research into clinical applications. Most models rely on rodents (rats and mice), whose living habits and spinal biomechanics differ fundamentally from humans. Common modeling approaches, such as needle puncture, autologous NP transplantation, laminectomy, and mechanical loading, primarily induce acute injury, whereas human IVDD typically develops progressively over years and is influenced by multifactorial risks, including genetics, aging, and mechanical stress. Additionally, widely used cell models fail to replicate the complex *in vivo* microenvironment, lacking contributions from immune cells, neurovascular networks, and full intercellular communication. Consequently, these models often inadequately reflect the comprehensive pathophysiological changes occurring during disease progression. While valuable for studying drug efficacy and mechanisms, they frequently overlook the complex interplay of factors driving IVDD [142].

As a result, existing models inadequately recapitulate the natural history of human IVDD and fail to fully elucidate the precise roles of PCDs in disease progression. To address these limitations, future research should prioritize aged or primate models and develop gradual, physiologically relevant modeling strategies that more accurately reflect human disease conditions (Table S5).

### 4.4 Future delivery strategies

Unlike metabolically active tissues, the IVD features a unique anatomical structure and a relatively isolated microenvironment, which limits the ability of conventional drug delivery approaches to achieve effective therapeutic concentrations at the lesion site [17]. Additionally, many natural products are inherently unstable and prone to spontaneous degradation under physiological conditions, necessitating specific modifications to maintain their pharmacological activity. Recent advances in biomaterials have enabled the development of novel delivery systems that facilitate targeted transport and sustained release of natural products, providing promising strategies to overcome these pharmaceutical limitations [143].

For example, transdermal hydrogel-based microneedle systems have been shown to significantly enhance the bioavailability of gastrodin and puerarin, while simultaneously mitigating first-pass metabolism and gastrointestinal side effects [144]. Similarly, the hyperbranched zwitterionic micelle system poly[2-(N-oxide-N,N-diethylamino)ethyl methacrylate]-poly(glycidyl methacrylate-ethylenediamine)-5-hexynoic acid@Cur@ginsenoside Rg3 (PPH@CR) effectively overcomes the poor water solubility and low bioavailability of Cur and ginsenoside Rg3, improving delivery efficiency and potentiating antitumor immune responses [145].

These advanced delivery strategies also show significant promise for IVDD treatment. For instance, fucoidan-loaded nanofibrous scaffolds can reconstruct the biomechanical microenvironment of degenerated discs while promoting macrophage M2 polarization, thereby suppressing ECM degradation, alleviating inflammation, and reducing oxidative stress [146]. Similarly, polyphenol-nucleic acid complexes exploit programmable delivery and antioxidant properties to restore NPC metabolic homeostasis and facilitate tissue regeneration [147].

Notably, gene therapy has emerged as a promising approach for IVDD. Nanotopology-enhanced RNA interference (RNAi) systems enable targeted silencing of senescence-related genes, rescuing NPCs from senescence and reversing disc degeneration [148]. Extracellular vesicles derived from MSCs deliver microRNAs (miRNAs) that regulate genes involved in ECM metabolism and inflammation, offering a cell-free gene regulatory strategy for IVDD [149]. Additionally, resveratrol trimer (caraphenol A) improves gene delivery efficiency by mitigating endosomal antiviral restrictions, providing a foundation for enhanced gene therapy efficacy [150]. Beyond gene therapy, natural products such as Cur and icariin exhibit synergistic therapeutic potential when combined with conventional drugs: Cur inhibits ferroptosis via the NRF2/GPX4 pathway, while icariin suppresses pyroptosis through the NRF2/HO-1 axis. Their combination with NSAIDs simultaneously targets cell death-mediated degeneration and alleviates pain [6,71]. Although adeno-associated virus (AAV) vectors have demonstrated potential for gene delivery in chronic diseases, their application in IVDD could facilitate precise modulation of gene expression. Collectively, these strategies, including precision gene therapies and natural product-drug combinations, offer promising avenues to overcome current challenges in IVDD treatment.

Notably, advanced delivery systems possess inherent synergistic potential when combined with PCD-targeting strategies, enabling precise modulation of cell death during IVDD [6]. The success of this combinatorial approach in fields such as oncology, inflammatory bowel disease, and osteoporosis [136] highlights its promising applicability to IVDD therapy, warranting further in-depth investigation.

## 5 Conclusion

Numerous natural products have shown significant potential in mitigating IVDD by modulating PCD-dependent mechanisms to enhance cellular viability within IVDs, offering promising new therapeutic avenues. Nonetheless, several challenges remain, including the precise quantification of PCD activity, identification of critical regulatory nodes, optimization of bioavailability and delivery strategies, mitigation of potential toxicity, and clinical validation of therapeutic efficacy. These hurdles collectively limit the clinical translation of natural product-based interventions. Nevertheless, as research continues to address these challenges, the prospect of applying natural products for IVDD treatment in clinical settings becomes increasingly attainable.

To achieve this goal, future research should focus on several key areas: i) elucidating the spatiotemporal overlap and dynamics of PCDs during IVDD progression, including variations in the intensity of specific PCD types across different pathological stages and the interactions among multiple PCDs within the same phase; ii) establishing comprehensive *in vitro* safety profiles and elucidating the toxicological mechanisms of natural products, while developing robust adverse reaction assessment and predictive systems for risk visualization; iii) advancing drug delivery systems tailored to the unique properties of natural products, such as designing carriers based on chemical classifications to enhance targeting specificity and *in vivo* stability; and iv) conducting rigorous, multicenter preclinical evaluations to ensure safety and efficacy prior to clinical translation. In conclusion, natural products possess substantial potential and versatile possibilities for modulating PCDs in IVDD, warranting further investigation and exploration.

## CRediT authorship contribution statement

**Songlin Liang:** Writing – original draft, Data curation. **Zhichao Li:** Methodology. **Renchang Chen:** Visualization. **Shang Gao:** Visualization. **Xintian Qu:** Investigation. **Chenmoji Wang:** Investigation. **Di Gao:** Investigation. **Hao Wang:** Software. **Jiatong Li:** Software. **Nianhu Li:** Writing – review & editing, Funding acquisition, Conceptualization.

## Declaration of competing interest

The authors declare that they have no known competing financial interests or personal relationships that could have appeared to influence the work reported in this paper.
