# Supplementary material for: Harnessing natural products to target programmed cell death for intervertebral disc degeneration: Mechanisms and opportunity
Source: J Pharm Anal. 2026 Feb 9;16(10):101583. doi: 10.1016/j.jpha.2026.101583 (PMC13696136; doi:10.1016/j.jpha.2026.101583)
Supplement: Supplementary file 1 [file mmc1.docx]

**Supplementary data**

**S1 Literature Search and Data Synthesis**

We conducted a comprehensive systematic search in PubMed, Cochrane Library, and Web of Science databases to identify natural products that mitigate IVDD through the regulation of PCDs. The search strategy employed the following query: *(Programmed Cell Death OR apoptosis OR autophagy OR ferroptosis OR pyroptosis OR necroptosis OR PANoptosis) AND (Intervertebral disc degeneration OR IVDD OR IDD OR Lumbar disc herniation)*. Additional continuous supplemental searches were performed using the following Medical Subject Headings (MeSH) and keywords: IVDD, intervertebral disc degeneration, low back pain, LBP, disc herniation, lumbar disc degeneration, and lumbar disc herniation. These terms were used individually or in combination with the following: natural product, traditional Chinese medicine, apoptosis, autophagy, ferroptosis, pyroptosis, necroptosis, and PANoptosis.

Inclusion criteria were limited to English-language publications (including original research articles, clinical trials, meta-analyses, and reviews) published between April 1, 2015, and April 1, 2025, that focused on the modulation of PCDs by natural products as a primary mechanism for alleviating IVDD. Studies were required to explicitly describe the therapeutic effects of natural products on IVDD via PCD regulation. The study selection process was independently performed by two investigators.

Through the screening process (Fig. S1), a total of 354 relevant publications were included in the full-text review. From these studies, 103 distinct natural products with demonstrated bioactivity against IVDD through PCD-related pathways were identified. We performed a systematic qualitative synthesis to extract and categorize mechanistic data, including molecular targets, signaling pathways, and therapeutic outcomes observed in experimental models and clinical studies (Fig. S1).

**S2. Searching strategy**

**Database: Pubmed**

**Search strategy:** (Programmed Cell Death or apoptosis or autophagy or ferroptosis or pyroptosis or necroptosis or PANoptosis) and (Intervertebral disc degeneration or IVDD or IDD or Lumbar disc herniation)

**Result（n=1429）：**

**Database: Cochrane**

**Search strategy:** (Programmed cell death OR Apoptosis OR Autophagy OR Ferroptosis OR Necroptosis OR Pyroptosis OR PANoptosis) AND (Intervertebral disc degeneration OR Lumbar disc herniation OR IVDD OR IDD)

**Result（n=4）：**

**Database: Web of Science**

**Search strategy:** (Programmed cell death OR Apoptosis OR Autophagy OR Ferroptosis OR Necroptosis OR Pyroptosis OR PANoptosis) AND (Intervertebral disc degeneration OR Lumbar disc herniation OR IVDD OR IDD)

**Result（n=1654）：**

Table S1. Natural products regulating apoptosis for IVDD

| Natural products | Structure | Experimental Models | Key pathways or targets | Optimal dose | Treatment effects | Ref. |
| --- | --- | --- | --- | --- | --- | --- |
| Acacetin |  | Rat NPCs, SD rats | Nrf2 pathway, MAPK pathway; HO-1, NQO1, SOD | *In vitro*: 0.3, 1 μM;  *In vivo*: 25 mg/kg | *In vitro*, alleviated TBHP-induced ROS generation, upregulated antioxidant proteins, relieved inflammation and ECM degradation. *In vivo*, ameliorated puncture-induced disc degeneration in rat tail model. | [1] |
| Acetylshikonin |  | Human NPCs, C57BL/6J mice | PI3K/Akt pathway; SOX4, Caspase-3, Bax | *In vitro*: 2 μM;  *In vivo*: 6 µL | Acetylshikonin delays IVDD and reduces LBP by inhibiting SOX4 expression via the PI3K/Akt pathway, which decreases NPCs apoptosis. | [2] |
| Allicin |  | Rat NPCs, SD rats | Caspase family; Bcl-2, Bax | *In vitro*: 20 mM;  *In vivo*: NA# (hydrogel) | Allicin, encapsulated in an ROS-responsive hydrogel, inhibited cell apoptosis, reduced oxidative stress and ECM degradation, and alleviated IVDD. | [3] |
| Alpinetin |  | Human NPCs | TLR4/MyD88 pathway; MMP3 | *In vitro*: 40 μM | Alpinetin repressed IL-1β-tempted apoptosis, inflammatory response, oxidative stress, and ECM degradation in NPCs through the TLR4/MyD88 pathway. | [4] |
| Amygdalin |  | Mice NPCs, C57BL/6J mice | NF-κB pathway; TNF-α, MMP13, COL2 | *In vitro*: 10 μM;  *In vivo*: 0.1 mg/kg; | Amygdalin alleviated pain symptoms and IDD progression by partly inhibiting NF-κB signaling. It prevented inflammatory factors release, endplate calcification, and chondrocyte degeneration. | [5] |
| Andrographolide |  | Rat NPCs | MAPK/Nrf2/HO-1 pathway; HO-1 | *In vitro*: 10 μM | Andrographolide inhibited static mechanical pressure-induced NPCs apoptosis and IDD by activating the MAPK/Nrf2/HO-1 pathway, inhibiting ROS accumulation and improving cell viability. | [6] |
|  |  | Human NPCs | NF-κB pathway | *In vitro*: 3, 6, 9 µg/ml | Andrographolide inhibited apoptosis and inflammation of degenerated NP cells, reversed NP degradation by blocking the NF-κB pathway, showing potential in IDD therapy. | [7] |
|  |  | Human NPCs | TLR4/MyD88/NF‑κB pathway, p65 | *In vitro*: 20 µM | Andrographolide alleviated IL-1β-induced apoptosis and ECM degradation in NP cells via inhibiting the TLR4/MyD88/NF-κB pathway, showing potential for IDD treatment. | [8] |
| Anisodamine |  | Human NPCs, SD rats | IL-6/JAK/STAT3 pathway; P53, P21 | *In vitro*: 10, 20, 40 μM;  *In vivo*: 1 mg/kg/day | Anisodamine inhibited the senescence and ECM degradation of NPCs by regulating the IL-6/JAK/STAT3 pathway, potentially improving the function of NPCs in IVDD. | [9] |
| Arctigenin |  | Human NPCs | miR-483-3p/NF-κB pathway | *In vitro*: 10, 50 µmol/L | Arctigenin inhibited apoptosis, ECM degradation, inflammation, and NF-κB pathway activation in IL-1β-induced HNPCs by up-regulating miR-483-3p, showing potential for IDD treatment. | [10] |
| Astragaloside IV |  | Human NPCs | PI3K/Akt pathway | *In vitro*: 100 µM | Astragaloside IV alleviates IL-1β-induced inflammation, apoptosis and ECM degeneration in HNPCs by activating the PI3K/Akt pathway. | [11] |
|  |  | Human NPCs，SD rats | NF-κB pathway | *In vitro*: 100 µM;  *In vivo*: 50 mg/kg/day | *In vitro*, AS IV alleviated IL-1β-induced inflammation, apoptosis, and ECM degeneration in NPCs by inhibiting the NF-κB pathway. *In vivo*, it alleviated puncture-induced IDD in rats. | [12] |
|  |  | Rat NPCs | TERT pathway | *In vitro*: 1, 3, 5, 10 mM | Astragaloside IV improves the viability and morphology of NPCs under HG conditions in a concentration-dependent manner, inhibits p16-mediated senescence and apoptosis. | [13] |
| Baicalein |  | Human NPCs | PI3K/Akt pathway; Bax, Cleaved Caspase-3, Bcl-2 | *In vitro*: 50 µM | Baicalein attenuated TNF-α-activated apoptosis in human NP cells through promoting the PI3K/Akt pathway, potentially attenuating disc degeneration. | [14] |
| Berberine |  | Human NPCs | NF-κB pathway; p65, IκBα, Bax | *In vitro*: 25 μM | Berberine protected human NP cells from IL-1β-induced apoptosis and ECM degradation by inhibiting the NF-κB pathway. | [15] |
| Chlorogenic acid |  | Mice NPCs, C57BL/6J mice | NF-κB pathway; MMP13, Aggrecan, Runx2 | *In vitro*: 0, 6.25, 12.5, 25 µM;  *In vivo*: 100 mg/kg | *In vivo*, chlorogenic acid suppressed NF-κB activity, mitigated cartilaginous endplate degeneration and IDD. *In vitro*, protected chondrocytes from catabolism and inflammation. | [16] |
| Curculigoside |  | Human NPCs, C57BL/6 mice | JAK/STAT3 pathway, BMAL1, Bax | *In vitro*: 10 µg/ml;  *In vivo*: 5 μM/kg/week | Curculigoside upregulated BMAL1 expression by inhibiting STAT3 phosphorylation, decreased NP cell apoptosis and alleviated IVDD. | [17] |
| Curcumin |  | Balb/C mice DRGs, primary neurons | AKT and ERK pathways; IL-6, COX-2, ROS | *In vitro*: 0.1, 1, 10 μM | Curcumin alleviated lumbar radiculopathy by reducing neuroinflammation, oxidative stress and nociceptive factors, and promoted disc regeneration. | [18] |
| Cyanidin-3-glucoside |  | Human NPCs | Nrf2/HO-1 pathway | *In vitro*: 12.5, 25, 50 μM | Cyanidin-3-glucoside treatment improved cell viability of HNPCs under HG condition, attenuated apoptosis and ECM degradation. | [19] |
| Cycloastragenol |  | Rat NPCs | TERT pathway | *In vitro*: 1, 3, 5, 10 mM | Cycloastragenol can inhibit HG-induced senescence and apoptosis of NPCs, activate telomerase, lengthen telomere length, increase cell viability, and improve cell morphology. | [13] |
| Daphnetin |  | Rat NPCs, C57BL/6 mice | Keap1/Nrf2/NF-κB axis, IL-6 | *In vitro*: 1, 2, 5 μM;  *In vivo*: 5 mg/kg | Daphnetin suppressed ROS generation, pro-inflammatory cytokines, and apoptosis, and delayed IVDD progression in mouse models. | [20] |
| Embelin |  | Human NPCs | PI3K/Akt pathway; Cleaved Caspase-3 | *In vitro*: 5, 10, 20 µM | Embelin protected NPCs from IL-1β -induced apoptosis and inflammation by activating the PI3K/Akt pathway. | [21] |
| Emodin |  | Rat NPCs, SD rats | LRP1, NF-ĸB pathway | *In vitro*: 5, 10, 20, 40, 80 μM;  *In vivo*: 10 μM | Emodin has therapeutic effect on IDD by inhibiting matrix metabolism disorder and apoptosis through LRP1 *in vitro* and *in vivo*, which was related to LRP1-mediated inhibition of NF-ĸB. | [22] |
|  |  | Rat NPCs, SD rats | LRP1-mediated NF-κB pathway; Bcl-2 | *In vitro*: 5, 10 μM;  *In vivo*: 10 μM (4 μL) | Emodin alleviated IDD by inhibiting matrix metabolism disorder and apoptosis through LRP1, which was related to LRP1-mediated inhibition of NF-κB. | [22] |
|  |  | Rat NPCs | NF-ĸB pathway | *In vitro*: 5, 10, 20, 40 μM | Emodin protected NPCs against IL-1β-induced apoptosis and inflammation via inhibiting ROS-mediated activation of NF-κB. | [23] |
| Engeletin |  | Rat NPCs, SD rats | NF-κB, MAPK, p65, p38 | *In vitro*: 80 µM;  *In vivo*: 50 µl (80 µM) | Engeletin effectively ameliorate the TNF-α-induced up-regulation of inflammatory mediators and MMP families, promoting the anabolism of ECM meanwhile. Cell apoptosis was also attenuated with the addition of engeletin. | [24] |
| Epigallocatechin-3-Gallate |  | Human NPCs | cGAS/Sting/NLRP3 pathway | *In vitro*: 5, 25 μM | Epigallocatechin-3-Gallate  treatment demonstrated significant protective effects in cell viability, apoptosis, cell cycle arrest and inflammatory status through down-regulation of cGAS/Sting/NLRP3 pathway. | [25] |
|  |  | Rat NPCs, SD rats | BMAL1-related circadian rhythm regulatory pathway | *In vitro*: 40 μM;  *In vivo*: 50 mg/kg | *In vitro*, EGCG attenuated H_2_O_2_-induced ECM degradation and apoptosis in NPCs. *In vivo*, it ameliorated IDD in a needle puncture rat model. | [26] |
| Evodiamine |  | Human NPCs | SIRT1/PI3K/Akt pathway | *In vitro*: 5, 10, 20 μM | Evodiamine inhibited LPS-induced NPCs apoptosis, ECM degradation and inflammation by upregulating SIRT1 and activating the PI3K/Akt pathway, protecting against IDD. | [27] |
| Fisetin |  | Rat NPCs | SIRT1, TNF-α, IL-6 | *In vitro*: 10 μM | Fisetin attenuated apoptosis and inflammation of NPMSCs induced by oxidative stress, and its effect might be related to SIRT1 pathway. | [28] |
| Fucoxanthin |  | Rat NPCs, SD rats | SIRT1-mediated PERK-eIF2α-ATF4-CHOP pathway; Bax | *In vitro*: 30 μM;  *In vivo*: 5, 10 mg/kg | Fucoxanthin alleviated ER stress, inhibited apoptosis and ECM degradation in NP cells, and ameliorated IDD in rats. | [29] |
| Ganoderic acid A |  | Rat NPCs | TLR4/NLRP3 pathway, Caspase-1 | *In vitro*: 4, 8 µM | Ganoderic acid A inhibited H_2_O_2_-induced apoptosis, inflammation, oxidative stress, and ECM degradation in NP cells by suppressing the TLR4/NLRP3 pathway, showing potential for IVDD treatment. | [30] |
| Genistein |  | Rat NPCs, SD rats | Nrf2, MMP13, Aggrecan | *In vitro*: 100 μM,  *In vivo*: 100 mg/kg/day | Genistein protected NPCs from oxidative stress-induced degeneration, inhibited apoptosis and ECM degradation, and ameliorated IDD in rats. | [31] |
| Genkwanin |  | Rat NPCs, SD rats | ITGA2/PI3K/Akt pathway, Bax, Bcl-2 | *In vitro*: 10, 20, 30, 40 µM;  *In vivo*: 20 mg/kg | Genkwanin  inhibits inflammation in NPCs, promotes ECM remodeling, suppresses cellular  senescence and apoptosis, through the ITGA2/PI3K/AKT, NF-κB and MAPK pathways. | [32] |
| Ginsenoside Rg1 |  | Rat NPCs, SD rats | NF-κB pathway; IL-1β, MMP3, Aggrecan | *In vitro*: 20, 50, 100 μM;  *In vivo*: 20, 40, 80 mg/kg/day | Ginsenoside Rg1 alleviated IVDD in rats and inhibited apoptosis, inflammatory response, and ECM degradation in IL-1β-induced NP cells via inhibiting NF-κB pathway. | [33] |
|  |  | Rat NPCs, SD Rats | COL2, cyclin D1, Wnt/β-catenin pathway | *In vivo*: 10, 20, 40 mg/kg/day;  *In vitro*: 20, 50, 100 µg/ml | Ginsenoside Rg1 could promote ECM synthesis of degenerative NPCs and inhibiting its apoptosis, improve the IVDD via inhibiting the Wnt/β-catenin pathway. | [34] |
| Ginsenoside Rg3 |  | Rat NPCs, SD rats | p38 MAPK pathway; Bax, Bcl-2, MMP3 | *In vitro*: 50 μg/mL;  *In vivo*: 20 mg/kg/day | Ginsenoside Rg3 showed anti-catabolic and anti-apoptotic effects, alleviated NPCs degeneration, and inhibited the p38 MAPK pathway in IL-1β-treated NPCs and IDD rats. | [35] |
| Glycyrrhizin |  | Human NPCs | p38/p-JNK pathway; HMGB1, Caspase-3/8, COL2 | *In vitro*: 100 µM | Glycyrrhizin suppressed inflammation and cell apoptosis via inhibiting HMGB1 through p38/p-JNK pathway, potentially alleviating IVDD. | [36] |
| Grape seed extract | NA# | New Zealand white rabbit | Bax, MMP13, Aggrecan | *In vivo*: 500 mg/kg | Grape seed extract had preventive and restorative effects on punctured-induced disc. It inhibited NPCs apoptosis and regulated the expression of ECM-related genes, thus showing potential in treating disc degenerative diseases. | [37] |
| Higenamine |  | Human NPCs | ROS-mediated PI3K/Akt pathway; Bcl-2, Bax, Caspase-3 | *In vitro*: 10, 20, 40 µM | Higenamine mitigated IL-1β-induced apoptosis in HNPCs in a dose-dependent manner; attenuated IL-1β-induced decrease of Bcl-2 and increase of Bax and cleaved caspase-3; repressed IL-1β-mediated increase of ROS level and activated the PI3K/Akt pathway. | [38] |
|  |  | Human NPCs | NF-κB pathway; iNOS, NO, PGE2, COX-2 | *In vitro*: 10, 20, 40 μM | Higenamine improved cell viability in IL-1β-induced NPCs; attenuated the up-regulation of inflammatory molecules and matrix-degrading enzymes; suppressed the IL-1β-induced activation of the NF-κB pathway. | [39] |
| Honokiol |  | Rat NPCs, SD rats | NLRP3, MMP13, COL2 | *In vitro*: 2.5, 5 μM;  *In vivo*: 30 mg/kg | Honokiol inhibited H_2_O_2_--induced apoptosis, oxidative stress and inflammation, and delayed IVDD progression, showing potential for IVDD treatment. | [40] |
| Hydroxysafflor yellow A |  | Rat NPCs | CA XII, MMP13, Bax/Bcl-2 | *In vitro*: 10 μM | Hydroxysafflor yellow A could attenuate oxidative stress injury and apoptosis induced by TBHP in the NP cell line, and improve the regulation of ECM balance. It increased the expression levels of COL2 and aggrecan, and decreased the expression level of MMP13. | [41] |
| Hyperoside |  | Human NPCs | SIRT1/NF-κB and Nrf2/ARE pathways | *In vitro*: 10, 20, 50 µM | Hyperoside ameliorated TNF-α-induced inflammation, ECM degradation and ER stress-mediated apoptosis in NPCs, potentially protecting against IDD. | [42] |
| Icariin |  | Human NPCs, SD rats | Nrf2pathway; NRF-1, TFAM | *In vitro*: 0.1, 1, 10 μM;  *In vivo*: 30 mg/kg | Icariin protected human NP cells from H_2_O_2_-induced apoptosis and ameliorated IVDD in rats by activating the Nrf2 pathway. | [43] |
| Irigenin |  | Human NPCs | MMPs, Caspase-3, COL2 | *In vivo*: 5 mg/kg | Irigenin suppressed apoptosis and ECM degradation in TNF-α-stimulated NP cells by reducing the expression of caspase-3 and MMPs. | [44] |
| Isoliquiritigenin |  | Rat NPCs, SD rats | PPARγ-dependent pathway, Bax | *In vitro*: 2.5, 5, 10 μM;  *In vivo*: 20, 40 mg/kg | Isoliquiritigenin effectively treated puncture-induced IVDD in rats. It reduced oxidative stress, restored mitochondrial function, inhibited NPCs apoptosis, and balanced ECM metabolism, thus slowing down the progression of IVDD. | [45] |
| Kaempferol |  | Human NPCs | MAPK pathway; Caspase-3, Bax | *In vitro*: 10 µM | Kaempferol protected NPCs from IL-1β -induced damages, like suppressing apoptosis, promoting cell viability and reducing ECM degradation. | [46] |
| Kukoamine A |  | Human NPCs | PI3K/Akt pathway, Bcl-2 | *In vitro*: 10, 20, 40 µM | Kukoamine A reduced LPS-induced apoptosis, ECM degradation, and inflammation in NPCs by activating the PI3K/Akt pathway, showing potential for IDD treatment. | [47] |
| Ligustilide |  | Rat NPCs, SD Rats | Caspase-3, Bax, COL2, MMPs, NF-κB pathway | *In vitro*: 2.5, 5, 10 μM  *In vivo*: 10 mg/kg/day | Ligustilide inhibits the inflammation response by inhibiting the NF-κB pathway. Moreover, the ligustilide could inhibit the apoptosis of NPCs and ameliorate the progression of IVDD in rat. | [48] |
| Lupeol |  | Rabbit NPCs | Bax, Bcl-2, Caspase-9 | *In vitro*: 60 μg/mL | Lupeol inhibited high–glucose-induced apoptosis in NPCs by enhancing anti-oxidative stress in mitochondria, potentially treating IVDD under hyperglycaemic conditions. | [49] |
| Luteolin |  | Human NPCs | SIRT6/NF-κB pathway; H3K9ac | *In vitro*: 1, 2, 4 μM | Luteolin alleviated TNF-α-induced inflammatory injury and senescence of HNPCs via the SIRT6/NF-κB pathway, showing potential for IDD treatment. | [50] |
| Luteoloside |  | Rat NPCs, SD Rats | iNOS, COX-2, TNF-α, IL-6, COL2, MMPs, Nrf2/HO-1, NF-κB pathway | *In vitro*: 2, 5, 10, 20 μM  *In vivo*: 10 mg/kg/day | Luteoloside maintains cell morphology and inhibits apoptosis in IL-1β-treated NPCs. It also suppresses inflammatory mediators and increases collagen II and aggrecan expression. | [51] |
| α-Mangostin |  | Human NPCs | NF-κB/NLRP3 pathway | *In vitro*: 40 µM | α-Mangostin protected NPCs from NLRP3 inflammasome-mediated apoptosis via the NF-κB pathway, showing potential for IDD prevention and treatment. | [52] |
| Marein |  | Human NPCs | NF-κB pathway, MMP, ROS, COL2 | *In vitro*: 30 μM | Marein protected HNPCs against HG-induced injury and ECM degradation, at least partly by inhibiting the ROS/NF-κB pathway. | [53] |
| Naringin |  | Rat NPMSCs | PI3K/Akt pathway; Bax, Bcl-2, cleaved caspase-3 | *In vitro*: 60 μM | Nar alleviated H_2_O_2_-induced NPMSC apoptosis and mitochondrial dysfunction via activating the ROS-mediated PI3K/Akt pathway. | [54] |
| Neochlorogenic acid |  | Rat BMSCs, SD rats | Nrf2/HO-1 pathway; NQO-1 | *In vitro*: 1.6 μM;  *In vivo*:2 μL with GelMA hydrogel | Neochlorogenic acid attenuated oxidative stress-induced damage in stem cells and reduced the apoptotic cell percentage. The GelMA+BMSC+Ncg combination significantly alleviated IVDD in rats. | [55] |
| Myricetin |  | Rat NPMSCs | SIRT1/PGC-1α pathway; Bax | *In vitro*: 50 μM | Myricetin protected rat NPMSCs from H_2_O_2_-induced apoptosis and senescence, and mitigated mitochondrial dysfunctions via the SIRT1/PGC-1α pathway. | [56] |
| Orientin |  | Rat NPCs, SD rats | AMPK/SIRT1 pathway | *In vitro*: 40 μM;  *In vivo*: 40 mg/kg | *In vitro*, Orientin increased SIRT1/AMPK in NPCs, maintained ECM and ER balance and decreased oxidative stress response. *In vivo*, Ori attenuated the pathological development of IVDD. | [57] |
| Oxymatrine |  | Rat NPCs, Kunming mice | MMP3, MMP9, COL2 | *In vitro*: 1 mg/mL;  *In vivo*: 10 μL | Oxymatrine liposomes could attenuate NP cell apoptosis, reduce the expression of MMP3/9 and IL-6, decrease COL2 degradation, and inhibit IVDD. | [58] |
| Panax notoginseng saponins | NA# | Human NPCs, SD rats | Akt/mTOR pathway; Caspase-3, MMP13 | *In vitro*: 1 μg/ml;  *In vivo*: 160, 200 mg/kg | Panax notoginseng saponins inhibited HNP cell apoptosis and proteoglycan degradation, alleviated disc degeneration in rats, potentially via Akt/mTOR pathway | [59] |
| Phillyrin |  | Rat NPCs, SD rats | NF-κB pathway; MMP3, BAX, Bcl-2 | *In vitro*: 10, 20 μM;  *In vivo*: 10 µM | Phillyrin alleviated IDD by reducing ROS production, inhibiting NF-κB pathway, suppressing ECM degradation and NPCs apoptosis. | [60] |
| Proanthocyanidins |  | Rat NPCs | PI3K/Akt pathway | *In vitro*: 10 µM | Proanthocyanidins inhibit IL-1β-induced apoptosis and aging of NP cells by activating the PI3K/Akt pathway. | [61] |
| Procyanidin B2 |  | Rat NPCs | Nrf2, PI3K/Akt-Nrf2 pathway | *In vitro*: 25 µg/ml, | Procyanidin B2 can alleviate the apoptosis of rat NPCs induced by oxidative stress, and it exerts its effect by activating the PI3K/Akt pathway and upregulating the expression of Nrf2. | [62] |
| Puerarin |  | Rat NPCs, SD rats | TLR4/NF-κB pathway | *In vitro*: 100, 200 μmol/mL;  *In vivo*: 100, 200 mg/kg/day | Puerarin can decrease ECM degradation and inflammation, inhibit apoptosis, and delay the progression of  IDD by inhibiting activation of the TLR4/NF-κB pathway. | [63] |
| Quercetin |  | Rat NPCs | PERK-eIF2α-ATF4 pathway; GRP78, CHOP, Caspase-12 | *In vitro*: 60 μM | Quercetin alleviated hyperglycemic-generated ER stress-induced apoptosis in NP cells via the PERK-eIF2α-ATF4 pathway, showing potential for IDD treatment. | [64] |
| *Raphanus Sativus* Linne extract (*Raphanus sativus* L.) | NA# | Human NPCs | TREM2, Bax, Bcl-2 | *In vitro*: 25, 100, 400 µg/mL | RSL protected NP cells from oxidative stress, regulated apoptosis-related proteins, increased ECM components, and inhibited ECM-degrading enzymes. | [65] |
| Resveratrol |  | Human NPCs | IL-6/JAK/STAT3 pathway; COL2 | *In vitro*: 10, 20,50 μM | Resveratrol improved NPCs viability, inhibited apoptosis and cycle arrest, protected from degeneration, regulated related protein levels. | [66] |
|  |  | Human NPCs | PI3K/Akt/mTOR/Caspase-3; PI3K/Akt/GSK-3β pathway | *In vitro*: 200 μM | Attenuated IL-1β-induced cell apoptosis, recovered cell viability; increased activated p-mTOR and p-GSK-3β, downregulated caspase-3; the anti-apoptosis process was not involved with activated p-NF-κB. | [67] |
| Rutin |  | Rat NPCs | Oxidative stress-related pathways; HSP70, Mn-SOD, Catalase | *In vitro*: 25, 50, 100, 200 μM | Rutin reduced ROS accumulation in TBHP-treated NPCs, increased antioxidant and matrix protein expressions, inhibited MMP13 expression. | [68] |
| Salvianolic acid B |  | Rat BMSCs, SD rats | JAK2-STAT3 pathway | *In vitro*: 10 μM (1% HAMA hydrogel);  *In vivo*: 10μM | *In vitro*, Salvianolic acid B decreased BMSCs apoptosis and promoted differentiation. *In vivo*, the combination of SalB, BMSCs, and 1% HAMA hydrogel alleviated disc degeneration with the best effect. | [69] |
| Sinapic acid |  | Rat NPCs, SD Rats | NO, PGE2, IL-6, TNF-α, Nrf2/HO-1 pathway | *In vitro*: 50, 100 μM;  *In vivo*: 20 mg/kg/day | Sinapic acid could protect the degeneration of NP cells and revealed the underlying mechanism of SA on Nrf2 activation in NP cells. | [70] |
| Sinomenine |  | Rat NPCs, SD rats | Keap1/Nrf2/NF-κB pathways; HO-1 | *In vitro*: 4 μM;  *In vivo*: 4 μM (5μL) | Sinomenine improved cell viability, reduced ROS levels and apoptosis. It alleviated IDD by activating Keap1/Nrf2 and inhibiting NF-κB pathways. | [71] |
| Sulforaphane |  | HNPCs, Rat NPCs, Lmna G609G/G609G mice | Progerin, MMP13,COL2 | *In vitro*: 5 μM;  *In vivo*: 10 mg/kg | Sulforaphane alleviated progerin-induced IDD by ameliorating aging defects and mitochondrial dysfunction, showing promise for IDD treatment. | [72] |
| Tyrosol |  | Human NPCs | SIRT1/PI3K/Akt pathway, MMP3 | *In vitro*: 10 μM | Tyrosol suppressed apoptosis and inflammation and regulated ECM remodeling in IL-1β-stimulated HNPCs by upregulating SIRT1 and activating the PI3K/Akt pathway. | [73] |

NA#: As this natural product is a mixture, it does not possess a defined chemical structure.

* The concentrations and doses are reported exactly as provided in the original referenced studies to accurately reflect the experimental conditions. A variety of units (e.g., μM, μg/mL, mg/kg) are therefore presented. This applies to all subsequent tables summarizing experimental data.

IVDD: Intervertebral disc degeneration; NPCs: Nucleus pulposus cells; Nrf2: Nuclear factor erythroid 2-related factor 2; MAPK: Mitogen-activated protein kinase; HO-1: Heme oxygenase-1; NQO1: NAD (P) H:quinone oxidoreductase 1; SOD: Superoxide dismutase; PI3K: Phosphatidylinositol 3-kinase; AKT: Protein kinase B; SOX4: SRY-related HMG-box 4; BAX: Bcl-2-associated X protein; Bcl-2: B-cell lymphoma 2; TLR4: Toll-like receptor 4; MyD88: Myeloid differentiation factor 88; MMPs: Matrix metalloproteinases; NF-κB: Nuclear factor kappa-B; TNF-α: Tumor necrosis factor-alpha; COL2: Collagen type II; p65: Nuclear factor kappa-B p65 subunit; IL-6: Interleukin-6; JAK: Janus kinase; STAT: Signal transducer and activator of transcription; TERT: Telomerase reverse transcriptase; IκBα: Inhibitor of nuclear factor kappa-B alpha; Runx2: Runt-related transcription factor 2; BMAL1: Brain and muscle ARNT-like 1; ERK: Extracellular regulated protein kinases; COX-2: Cyclooxygenase-2; ROS: Reactive oxygen species; Keap1: Kelch-like ECH-associated protein 1; LRP1: Low-density lipoprotein receptor-related protein 1; cGAS: Cyclic GMP-AMP synthase; STING: Stimulator of interferon genes; NLRP: NOD-like receptor family pyrin domain-containing; PERK: Protein kinase R-like endoplasmic reticulum kinase; eIF2α: Eukaryotic translation initiation factor 2 alpha; ATF4: Activating transcription factor 4; CHOP: CCAAT/enhancer binding protein homologous protein; SIRT1: Sirtuin 1; ITGA2: Integrin alpha 2; Wnt β-catenin: Wnt and β-catenin signaling pathway; p38: p38 mitogen-activated protein kinase; p-JNK: Phosphorylated c-Jun N-terminal kinase; HMGB1: High mobility group box 1; iNOS: Inducible nitric oxide synthase; NO: Nitric oxide; PGE2: Prostaglandin E2; CA XII: Carbonic anhydrase XII; TFAM: Mitochondrial transcription factor A; PPARγ: Peroxisome proliferator-activated receptor gamma; H3K9ac: Histone H3 lysine 9 acetylation; PGC-1α: Peroxisome proliferator-activated receptor gamma coactivator 1-alpha; AMPK: Adenosine monophosphate-activated protein kinase; mTOR: Mammalian target of rapamycin; GSK-3β: Glycogen synthase kinase-3 beta; HSP70: Heat shock protein 70; GRP78: Glucose-regulated protein 78; TREM2: Triggering receptor expressed on myeloid cells 2.

Table S2. Natural products regulating autophagy for IVDD

| Natural products | Structure | Experimental Models | Key pathways or targets | Optimal dose | Treatment effects | Ref. |
| --- | --- | --- | --- | --- | --- | --- |
| Achyranthoside D |  | Rat NPCs, SD rats | PI3K/Akt/mTOR pathway; LC3, P62, COL2 | *In vitro*: 40 μg/mL;  *In vivo*: 60, 120, 240 μg/g/day | Achyranthoside D alleviated IVD tissue injury and NPCs damage; regulated autophagy and PI3K/Akt/mTOR pathway; increased glycoprotein secretion and inhibited apoptosis. | [74] |
| Andrographolide |  | Human NPCs, SD rats | miR-9/FoxO3/PINK1/Parkin pathway | *In vitro*: 3, 6, 9 μg/mL;  *In vivo*: 150 mg/kg | Andrographolide improved NPCs proliferation and autophagy, inhibited apoptosis and oxidative stress, alleviated IDD pathological changes. | [75] |
| Apigenin |  | Rat NPCs, SD rats | AMPK/mTOR pathway, TFEB, LC3, p62 | *In vitro*: 10, 25, 50 μM;  *In vivo*: 10 mg/kg | Apigenin inhibited TBHP-induced apoptosis, senescence and ECM degradation in NP cells, restored autophagic flux and ameliorated IVDD progression in rats. | [76] |
| Apigetrin |  | Rat NPCs, SD Rats | ATG, LC3, NF-κB, PI3K/Akt/mTOR, MAPK pathway | *In vitro*: 50 μM;  *In vivo*: 50 μM | Apigetrin is capable of alleviating IVDD by inhibiting inflammation and balancing disturbed ECM homeostasis. | [77] |
| Berberine |  | Rat NPCs, SD rats | Bax, Bcl-2, LC3 | *In vitro*: 25 μM;  *In vivo*: 150 mg/kg | Berberine inhibited NP cell apoptosis and ECM degradation; activated autophagy; ameliorated disc degeneration in a rat model. | [78] |
|  |  | Rat NPCs, SD rats | IRE1/JNK pathway; GRP78, CHOP, LC3 | *In vitro*: 1, 2, 4, 8 μM;  *In vivo*: 150 mg/kg/day | Berberine could modulate autophagy. It decreased the conversion of LC3-I to LC-3 II and Beclin-1 levels, increased p62, and reduced autophagosomes and autolysosomes. | [79] |
| Curcumin |  | Human CEPCs, SD rats | Beclin-1, LC3-II, Cleaved caspase-3 | *In vitro*: 20 μM;  *In vivo*: 50 mg/kg | Curcumin upregulated autophagy, reduced phenotype loss of endplate chondrocytes, relieved IVDD. | [80] |
|  |  | Rat CEPCs | Bax, Bcl-2 | *In vitro*: 10, 15, 20 μM | Curcumin promoted proliferation of IL-1β-treated chondrocytes, inhibited apoptosis, downregulated Bax expression, and upregulated Bcl-2 expression. | [81] |
| Cyanidin |  | Rat NPCs, SD rats | JAK2/STAT3 signal pathway, Aggrecan, COL2, MMP13, LC3 | *In vitro*: 25, 50 μM;  *In vivo*: 50 mg/kg | Inhibited apoptosis and ECM degradation of IL-1β-induced NPCs, promoted autophagy. *In vivo*, it alleviated disc degeneration in IVDD rats. | [82] |
| 6-gingerol |  | Human NPMSCs | PI3K/Akt pathway; Beclin-1, LC3-II, p62 | *In vitro*: 30 μM | 6-gingerol decreased intracellular ROS levels, inhibited NPMSC apoptosis, promoted ECM expression, activated autophagy. | [83] |
| Isoginkgetin |  | Human NPCs, SD rats | Beclin-1, ATG7, LC3 | *In vitro*: 20 μM;  *In vivo*: 20 μM | IGK@SeNP scavenged ROS, enhanced autophagy in NPCs, inhibited ECM degradation and cell apoptosis, and alleviated IVDD in rats. | [84] |
| Isorhapontigenin |  | Rat NPCs, SD Rats | P53, P21, P16, COL2, LC3, PI3K/Akt/mTOR pathway | *In vitro*: 10, 20 μM;  *In vivo*: 40 mg/kg | Isorhapontigenin delays aging and ECM degradation by promoting PI3K/Akt/mTOR-mediated autophagy. | [85] |
| Lycopene |  | Human NPCs | Nrf2 pathway, Nrf2, Bax, Bcl2 | *In vitro*: 5 μM | Lycopene promote Nrf2 expression and autophagy while reducing ECM degradation in NPCs under oxidative stress. | [86] |
| Moracin M |  | Rat NPCs | PI3K/Akt/mTOR pathway; IL-1β, TNF-α, LC3 | *In vitro*: 5, 10,  20 μM | Moracin M inhibited LPS-induced inflammation in NPCs; regulated gene expressions of MMPs, aggrecan and COL2; promoted autophagy. | [87] |
| Myricetin |  | Rat NPCs, SD rats | JAK2/STAT3 pathway, Bax, Bcl-2, LC3 | *In vitro*: 10, 20 μM;  *In vivo*: 20 mg/kg/day | Myricetin mitigated IL-1β-induced NPCs apoptosis and ECM degradation, enhanced autophagy, and alleviated IDD by inhibiting the JAK2/STAT3 pathway. | [88] |
| Naringin |  | Rat NPCs, SD rats | AMPK-autophagy pathway; LC3, Beclin-1, Caspase-3 | *In vitro*: 25, 50, 100 μM;  *In vivo*: 80 mg/kg | Naringin protected NP cells from apoptosis by activating autophagy via the AMPK pathway and ameliorated IDD in rats. | [89] |
| Physalin A |  | Rat NPCs, SD rats | PI3K/Akt/mTOR pathway; p-P65, LC3, α-SMA | *In vitro*: 20 μM;  *In vivo*: 20 μM (2 μl) | Physalin A relieved IVDD progression via anti-inflammation, fibrosis reduction, and autophagy enhancement. Improved ECM metabolism and inflammatory environment in NP cells. | [90] |
| Procyanidin C1 |  | Human NPCs, SD rats | SIRT3/FOXO3 pathwaypathway | *In vitro*: 10, 20, 40 μM;  *In vivo*: 8 mg/kg | Ameliorated acidic pH stress induced NP degeneration, alleviated apoptosis and excessive mitophagy, and attenuated rat IVDD. | [91] |
| Quercetin |  | Human NPCs, Rat NPCs, SD rats | SIRT1-autophagy pathway; LC3, Caspase-3 | *In vitro*: 60 μM;  *In vivo*: 100 mg/kg | Quercetin protected NP cells from apoptosis and attenuated ECM degeneration via the SIRT1-autophagy pathway, alleviating IDD in rats. | [92] |
| Resveratrol |  | Human NPCs, New Zealand rabbits | LC3, p62 | *In vitro*: 50 μM;  *In vivo*: 50 μM | Resveratrol alleviated mitochondrial dysfunction and cell apoptosis under oxidative stress, delayed the progression of IVDD by activating autophagy, and improved IVDD in rabbits *in vivo*. | [93] |
| Scutellarin |  | Rat NPCs | PI3K/PTEN/Akt-ATG5-Rab8a pathway; ATG5, Rab8a | *In vitro*: 20 μM | Scutellarin elevated autophagy levels in NPCs, upregulated Rab8a, promoted exosome release, potentially preventing IDD. | [94] |
| Sinomenine |  | Rat NPCs, SD rats | Beclin-1, ATG5, Bax | *In vitro*: 3.33 mM;  *In vivo*: 25, 75 mg/kg | Sinomenine exerted an anti-apoptotic effect against oxidative stress  in rat NP cells by promoting autophagy. | [95] |
| Spatholobi Caulis | NA# | Rat NPCs, SD rats | DAPK1, ATG7, Bcl-2 | *In vitro*: 12.5, 25, 50, 100 μg/mL;  *In vivo*: 500 μg/mL（QCS-OST/SC hydrogel） | Spatholobi Caulis enhanced cell viability, promoted autophagy, inhibited apoptosis and senescence of NPCs, regulated ECM-related gene expression, and alleviated disc degeneration in rats. | [96] |
| Tetrandrine |  | Human NPCs, SD rats | Aggrecan, COL2, MMP13 | *In vitro*: 0.1, 0.5, 1 μM;  *In vivo*: 40 mg/kg | Tetrandrine inhibited oxidative stress, apoptosis and ECM degradation in TBHP-treated NP cells and delayed rat IDD by promoting autophagy. | [97] |
| Trigonochinene E |  | Human NPCs | PERK-calcineurin axis, IRE1α-STAT3 axis; TFEB, TFE3, LC3 | *In vitro*: 40 μM | TE promoted lysosomal biogenesis and autophagy in cells. It protected NP cells from oxidative stress and ameliorated IVDD-related features *in vitro*. | [98] |
| Urolithin A |  | Rat NPCs, SD rats | LC3, p62, Caspase-3 | *In vitro*: 2, 20 μM;  *In vivo*: 25 mg/kg/day | Urolithin A activates mitophagy in NP cells, inhibits apoptosis, and alleviates IVDD in rats. | [99] |

NA#: As this natural product is a mixture, it does not possess a defined chemical structure.

*Units are reported as in the original studies. See Table S1 for details.

IVDD: Intervertebral disc degeneration; NPCs: Nucleus pulposus cells; PI3K: Phosphatidylinositol 3-kinase; AKT: Protein kinase B; mTOR: Mammalian target of rapamycin; LC3: Microtubule-associated protein 1 light chain 3; COL2: Collagen type II; FOXO3: Forkhead box O3; PINK1: PTEN-induced kinase 1; IDD: Intervertebral disc; AMPK: Adenosine monophosphate-activated protein kinase; TFEB: Transcription factor EB; TBHP: Tert-butyl hydroperoxide; ECM: Extracellular matrix; ATG: Autophagy-related gene; NF-κB: Nuclear factor kappa-B; MAPK: Mitogen-activated protein kinase; BAX: Bcl-2-associated X protein; Bcl-2: B-cell lymphoma 2; IRE1: Inositol-requiring enzyme 1; JNK: c-Jun N-terminal kinase; GRP78: Glucose-regulated protein 78; CHOP: CCAAT/enhancer binding protein homologous protein; JAK2: Janus kinase 2; STAT3: Signal transducer and activator of transcription 3; MMP13: Matrix metalloproteinase 13; IL-1β: Interleukin-1 beta; TNF-α: Tumor necrosis factor-alpha; p-P65: Phosphorylated nuclear factor kappa-B p65 subunit; α-SMA: Alpha-smooth muscle actin; SIRT: Sirtuin; PTEN: Phosphatase and tensin homolog; Rab8a: Ras-related protein Rab-8A; DAPK1: Death-associated protein kinase 1; PERK: Protein kinase R-like endoplasmic reticulum kinase; TFE3: Transcription factor E3.

Table S3. Natural products regulating ferroptosis for IVDD

| Natural products | Structure | Experimental Models | Key pathways or targets | Optimal dose | Treatment effects | Ref. |
| --- | --- | --- | --- | --- | --- | --- |
| Astaxanthin |  | Mice CEPCs, C57BL/6 mice | Nrf2/HO- 1 pathway, GPX4, Bax | *In vitro*: 5, 10, 20, 40, 80 μM;  *In vivo*: 30 mg/kg | Astaxanthin activated Nrf2/HO-1 pathway, inhibited oxidative stress, ferroptosis and apoptosis, moderated chondrocytes and ECM homeostasis, and suppressed CEP calcification. | [100] |
| Cynarin |  | Rat NPCs, SD rats | Nrf2, GPX4, MMP13 | *In vitro*: 50 μM;  *In vivo*: 25, 50 μM | Cynarin inhibited TNF-α-induced ferroptosis in NPCs, upregulated the expression levels of Nrf2 and GPX4 in NPCs, and mitigated the severity of puncture-induced IVDD and the expression level of GPX4 in tissues *in vivo*. | [101] |
| Fisetin |  | Rat NPCs, SD rats | Nrf2/HO-1 axis, GPX4 | *In vitro*: 20 μM;  *In vivo*: 20 mg/kg/day | Fisetin inhibited ferroptosis, attenuated ECM degradation and senescence, and mitigated IVDD. | [102] |
| Gallic acid |  | Rat NPCs, SD Rats | P53, NRF2, GPX4, ACSL4, HO-1, SLC7A11, FTH-1, ADAMTS | *In vitro*:20, 50, 100 μM;  *In vivo*: 25, 50, 100 mg/kg | Gallic acid treats IDD by reducing the degradation of ECM and pathological changes in IDD. It can also mitigate ferroptosis by reducing oxidative stress and lipid peroxidation in rat NPCs. | [103] |
| Hesperidin |  | Human NPCs, C57BL/6 mice | Nrf2/NF-κB axis, P65, GPX4 | *In vitro*: 1, 5, 10, 20, 50, 100 μM;  *In vivo*: 50 mg/kg | Hesperidin ameliorated ECM degradation and inflammatory response, had anti-oxidative stress effect, suppressed ferroptosis and NF-κB pathway activation. | [104] |
| Icariin |  | Mice CEPCs, C57BL/6 mice | Nrf2/HO-1 pathway, Parkin, GPX4 | *In vitro*: 0.1, 1, 10 μM;  *In vivo*: 30 mg/kg | Icariin alleviated CEP degeneration and IVDD progression, suppressed chondrocytes apoptosis, ECM degradation, and ferroptosis. | [105] |
| Pinocembrin |  | Mice CEPCs, C57BL/6 mice | Nrf2 pathway, Nrf2, Parkin, GPX4 | *In vitro*: 50 μM;  *In vivo*: 20 mg/kg | Pinocembrin ameliorated cartilage endplate degeneration and IDD progression, suppressed ECM degradation, apoptosis, and oxidative stress-induced calcification. | [106] |
| Schisantherin A |  | Huamn NPCs,  C57BL/6 mice | LOX-1/NF-κB/ NOX signal | *In vivo*: 40 mg/kg;  *In vitro*: 5 μM | Schisantherin A improved ferroptosis-related indicators and alleviated ferroptosis-induced degenerative changes in NPCs. | [107] |

*Units are reported as in the original studies. See Table S1 for details.

IVDD: Intervertebral disc degeneration; CEPCs: Cartilage endplate cells; Nrf2: Nuclear factor erythroid 2-related factor 2; HO-1: Heme oxygenase-1; GPX4: Glutathione peroxidase 4; BAX: Bcl-2-associated X protein; ECM: Extracellular matrix; CEP: Cartilage endplate; MMP13: Matrix metalloproteinase 13; ACSL4: Acyl-CoA synthetase 4; SLC7A11: Solute carrier family 7 member 11; FTH-1: Ferritin heavy chain 1; ADAMTS: A disintegrin and metalloproteinase with thrombospondin motifs; IDD: Intervertebral disc; NF-κB: Nuclear factor kappa-B; LOX-1: Lectin-like oxidized low-density lipoprotein receptor 1; NOX: Nicotinamide adenine dinucleotide phosphate oxidase.

Table S4. Natural products regulating pyroptosis for IVDD

| Natural products | Structure | Experimental Models | Key pathways or targets | Optimal dose | Treatment effects | Ref. |
| --- | --- | --- | --- | --- | --- | --- |
| Baicalin |  | Human NPCs | MAPK pathway, MMP3, MMP13, ADAMTS5 | *In vitro*: 25, 50, 100 μM | Baicalin attenuated IL-1β -induced reduction in cell viability, inflammation, oxidative stress, and ECM degradation in NPCs. | [108] |
| Ginkgetin |  | Rat NPCs, SD rats | NLRP3 inflammasome pathway; NLRP3, Caspase-1, IL-1β | *In vitro*: 10, 20, 40 μM;  *In vivo*: 100 mg/kg | Ginkgetin increased NPCs viability, inhibited apoptosis, reduced ECM degradation, attenuated inflammation, inactivated NLRP3 inflammasome. | [109] |
| Magnoflorine |  | THP-1 cells, Human NPCs | HMGB1/MyD88/NF-κB pathway and NLRP3 inflammasome | *In vitro*: 12.5, 25, 50, 100 μg/ml | Magnoflorine alleviates "M1" polarized macrophage-mediated NP cell damage, dampens HMGB1 expression, and inactivates the MyD88/NF-κB pathway and NLRP3 inflammasome in NP cells. | [110] |
| Maltol |  | Rat NPCs,  Mice | PI3K/Akt/NF-κB pathway; NLRP3, p65 | *In vitro*: 5, 10, 20 μM;  *In vivo*: 15, 30 mg/kg | Maltol ameliorated IVDD, inhibited ECM degradation and inflammatory response, regulated pyroptosis. | [111] |
| Morin |  | Rat NPCs, C57BL/6 mice | TXNIP/NLRP3/Caspase-1/IL-1β pathway; NLRP3, Caspase-1 | *In vitro*: 12.5, 25, 50, 100 nM;  *In vivo*: 30 mg/kg | Morin inhibited pyroptosis of NP cells, ameliorated IVDD, increased expression of COL2 and aggrecan, decreased expression of IL-1β. | [112] |
| Naringin |  | Mice CEPCs, C57BL/6J mice | SIRT3/FOXO3a/Parkin pathway, NLRP3, Parkin | *In vitro*: 50 μM;  *In vivo*: 50 mg/kg | Naringin mitigated IVDD progression and CEP calcification, inhibited NLRP3 inflammasome activation, promoted mitophagy, and suppressed apoptosis and ECM degradation. | [113] |
| Notoginsenoside R1 |  | Human NPCs, SD Rats | NF-κB/NLRP3 pathways, IL-1β, | *In vitro*: 10, 25, 50 μM;  *In vivo*: 50 mg/kg | Notoginsenoside R1 inactivated NF-κB/NLRP3 pathways, improved cellular functions of NPCs, and suppressed cell pyroptosis. | [114] |
| Paeoniflorin |  | Rat NPCs, SD rats | NLRP3, ASC, GSDMD-N | *In vitro*: 200 μM;  *In vivo*: 20 mg/kg | Paeoniflorin alleviated IVDD, inhibited pyroptosis of NPCs, reduced lactic acid accumulation and matrix degradation. | [115] |
| Rhein |  | Rat NPCs, SD rats | NLRP3, Caspase-1, GSDMD | *In vitro*: 25 µM;  *In vivo*: 5 µL | Rhein improved inflammatory microenvironment, regulated ECM metabolism, inhibited NLRP3 inflammasome aggregation and cell pyroptosis, and delayed IVDD. | [116] |
| Scutellarin |  | Human NPCs, SD rats | NF-κB and MAPK pathway;  NLRP3, COX-2, iNOS | *In vitro*: 1 μM;  *In vivo*: 100 ng/μl | Scutellarin attenuated inflammatory reaction, retained ECM production, suppressed apoptosis, reversed oxidative stress and mitochondrial dysfunction, and alleviated IVD degeneration. | [117] |
| Sinapine thiocyanate |  | Rat NPCs, SD rats | JAK1/STAT3 pathway, NLRP3, Caspase-1, p20, IL-1β, IL-18 | *In vitro*: 25, 50, 75 mg/L;  *In vivo*: 4,8 mg/kg | Sinapine thiocyanate repressed LPS-induced pyroptosis by inhibiting NLRP3, IL-1β, and IL-18 in NPCs. Alleviated IDD *in vivo*, inhibited the activation of JAK1/STAT3, and restrained NLRP3-mediated pyroptosis. | [118] |

*Units are reported as in the original studies. See Table S1 for details.

IVDD: Intervertebral disc degeneration; NPCs: Nucleus pulposus cells; MAPK: Mitogen-activated protein kinase; MMP: Matrix metalloproteinases; ADAMTS5: A disintegrin and metalloproteinase with thrombospondin motifs 5; NLRP3: NOD-like receptor family pyrin domain-containing 3; IL: Interleukin; HMGB1: High mobility group box 1; MyD88: Myeloid differentiation factor 88; NF-κB: Nuclear factor kappa-B; PI3K: Phosphatidylinositol 3-kinase; AKT: Protein kinase B; p65: Nuclear factor kappa-B p65 subunit; TXNIP: Thioredoxin-interacting protein; SIRT3: Sirtuin 3; FOXO3a: Forkhead box O3a; ASC: Apoptosis-associated speck-like protein containing a CARD; GSDMD-N: N-terminal fragment of gasdermin D; GSDMD: Gasdermin D; COX-2: Cyclooxygenase-2; iNOS: Inducible nitric oxide synthase; JAK1: Janus kinase 1; STAT3: Signal transducer and activator of transcription 3

Table S5. Bridging the Gap: From Natural Products to Clinically Approved Therapies for IVDD

| Aspect | Natural Products | Approved Drugs | Development Strategy |
| --- | --- | --- | --- |
| Chemical Properties | Broad resources, structural diversity, novel scaffolds | Defined structure, high purity, batch consistency | Purification, structural modification |
| Biological Activity | Multi-target effects, synergy, holistic modulation | Target specificity, well-defined mechanisms | Target identification, mechanistic validation |
| Therapeutic Efficacy | Efficacy in cellular/animal models, innovative therapeutic concepts | Proven efficacy in human clinical trials | Advancement to clinical studies |
| Source & Development | Natural origin, high biocompatibility, lead compound potential | Scalable production, stringent quality control | Process optimization, quality standard establishment |

Fig. S1 Literature screening process

**References**

[1] H. Wang, Z. Jiang, Z. Pang, et al., Acacetin alleviates inflammation and matrix degradation in nucleus pulposus cells and ameliorates intervertebral disc degeneration *in vivo*, Drug Des. Dev. Ther. 14 (2020) 4801–4813.

[2] Y. Huang, L. Lei, Z. Zhao, et al., Acetylshikonin promoting PI3K/Akt pathway and inhibiting SOX4 expression to delay intervertebral disc degeneration and low back pain, J. Orthop. Res. 42 (2024) 172–182.

[3] T. Zhang, Q. Huang, L. Lu, et al., ROS-responsive hydrogel loaded with allicin suppresses cell apoptosis for the treatment of intervertebral disc degeneration in a rat model, World Neurosurg. 193 (2025) 675–686.

[4] J. Xia, D. Jia, J. Wu, Protective effects of alpinetin against interleukin-1β-exposed nucleus pulposus cells: Involvement of the TLR4/MyD88 pathway in a cellular model of intervertebral disc degeneration, Toxicol. Appl. Pharmacol. 492 (2024), 117110.

[5] Q. Zeng, Q. Sun, H. Xu, et al., Amygdalin delays cartilage endplate degeneration and improves intervertebral disc degeneration by inhibiting NF-κB signaling pathway and inflammatory response, J. Inflamm. Res. 16 (2023) 3455–3468.

[6] C. Zhang, Z. Lu, C. Lyu, et al., Andrographolide inhibits static mechanical pressure-induced intervertebral disc degeneration via the MAPK/Nrf2/HO-1 pathway, Drug Des. Dev. Ther. 17 (2023) 535–550.

[7] J. Liu, T. Jiang, M. He, et al., Andrographolide prevents human nucleus pulposus cells against degeneration by inhibiting the NF-κB pathway, J. Cell. Physiol. 234 (2019) 9631–9639.

[8] L. Zhang, Q. Chen, H. Wang, et al., Andrographolide mitigates IL‑1β‑induced human nucleus pulposus cells degeneration through the TLR4/MyD88/NF‑κB signaling pathway, Mol. Med. Rep. 18 (2018) 5427–5436.

[9] N. Tang, Y. Dong, C. Chen, et al., Anisodamine maintains the stability of intervertebral disc tissue by inhibiting the senescence of nucleus pulposus cells and degradation of extracellular matrix via interleukin-6/Janus kinases/signal transducer and activator of transcription 3 pathway, Front. Pharmacol. 11 (2020), 519172.

[10] Z. Ji, R. Guo, Z. Ma, et al., Arctigenin inhibits apoptosis, extracellular matrix degradation, and inflammation in human nucleus pulposus cells by up-regulating miR-483-3p, J. Clin. Lab. Anal. 36 (2022), e24508.

[11] L. Zhang, J. Gao, Z. Li, et al., Astragaloside IV relieves IL-1β-induced human nucleus pulposus cells degeneration through modulating PI3K/Akt signaling pathway, Medicine 102 (2023), e34815.

[12] Y. Tian, X. Chu, Q. Huang, et al., Astragaloside IV attenuates IL-1β-induced intervertebral disc degeneration through inhibition of the NF-κB pathway, J. Orthop. Surg. Res. 17 (2022), 545.

[13] H. Hong, J. Xiao, Q. Guo, et al., Cycloastragenol and Astragaloside IV activate telomerase and protect nucleus pulposus cells against high glucose-induced senescence and apoptosis, Exp. Ther. Med. 22 (2021), 1326.

[14] Y. Liu, D. Liu, Z. Wang, et al., Baicalein alleviates TNF-α-induced apoptosis of human nucleus pulposus cells through PI3K/AKT signaling pathway, J. Orthop. Surg. Res. 18 (2023), 292.

[15] L. Lu, J. Hu, Q. Wu, et al., Berberine prevents human nucleus pulposus cells from IL‑1β‑induced extracellular matrix degradation and apoptosis by inhibiting the NF‑κB pathway, Int. J. Mol. Med. 43 (2019) 1679–1686.

[16] Q. Ge, J. Ying, Z. Shi, et al., Chlorogenic Acid retards cartilaginous endplate degeneration and ameliorates intervertebral disc degeneration via suppressing NF-κB signaling, Life Sci. 274 (2021), 119324.

[17] L. Lei, H. Wang, Z. Zhao, et al., Curculigoside upregulates BMAL1 to decrease nucleus pulposus cell apoptosis by inhibiting the JAK/STAT3 pathway, Osteoarthr. Cartil. 33 (2025) 412–425.

[18] L. Xiao, M. Ding, A. Fernandez, et al., Curcumin alleviates lumbar radiculopathy by reducing neuroinflammation, oxidative stress and nociceptive factors, Eur. Cells Mater. 33 (2017) 279–293.

[19] X. Bai, Y. Lian, C. Hu, et al., Cyanidin-3-glucoside protects against high glucose-induced injury in human nucleus pulposus cells by regulating the Nrf2/HO-1 signaling, J. Appl. Toxicol. 42 (2022) 1137–1145.

[20] S. Chen, Y. Huang, L. Lei, et al., Daphnetin ameliorates intervertebral disc degeneration via the Keap1/Nrf2/NF-κB axis *in vitro* and *in vivo*, Int. Immunopharmacol. 145 (2025), 113785.

[21] X. Bai, J. Wang, S. Ding, et al., Embelin protects against apoptosis and inflammation by regulating PI3K/Akt signaling in IL-1β-stimulated human nucleus pulposus cells, Tissue Cell 82 (2023), 102089.

[22] D. Yao, M. Li, K. Wang, et al., Emodin ameliorates matrix degradation and apoptosis in nucleus pulposus cells and attenuates intervertebral disc degeneration through LRP1 *in vitro* and *in vivo*, Exp. Cell Res. 432 (2023), 113794.

[23] X. Zhu, S. Guo, M. Zhang, et al., Emodin protects against apoptosis and inflammation by regulating reactive oxygen species-mediated NF-κB signaling in interleukin-1β-stimulated human nucleus pulposus cells, Hum. Exp. Toxicol. 42 (2023), 09603271221138552.

[24] B. Li, X. Yang, P. Zhang, et al., Engeletin alleviates the inflammation and apoptosis in intervertebral disc degeneration via inhibiting the NF-κB and MAPK pathways, J. Inflamm. Res. 15 (2022) 5767–5783.

[25] Y. Tian, Z. Bao, Y. Ji, et al., Epigallocatechin-3-gallate protects H2O2-induced nucleus pulposus cell apoptosis and inflammation by inhibiting cGAS/sting/NLRP3 activation, Drug Des. Dev. Ther. 14 (2020) 2113–2122.

[26] L. Mei, Y. Zheng, T. Ma, et al., (-)-epigallocatechin-3-gallate ameliorates intervertebral disc degeneration through reprogramming of the circadian clock, Front. Pharmacol. 12 (2021), 753548.

[27] J. Kuai, N. Zhang, Upregulation of SIRT1 by Evodiamine activates PI3K/AKT pathway and blocks intervertebral disc degeneration, Mol. Med. Rep. 26 (2022), 265.

[28] Q. Zhou, C. Zhu, A. Xuan, et al., Fisetin regulates the biological effects of rat nucleus pulposus mesenchymal stem cells under oxidative stress by sirtuin-1 pathway, Immun. Inflamm. Dis. 11 (2023), e865.

[29] C. Teng, J. Wu, Z. Zhang, et al., Fucoxanthin ameliorates endoplasmic reticulum stress and inhibits apoptosis and alleviates intervertebral disc degeneration in rats by upregulating Sirt1, Phytother. Res. 38 (2024) 2114–2127.

[30] D. Wang, X. Cai, F. Xu, et al., Ganoderic Acid A alleviates the degeneration of intervertebral disc via suppressing the activation of TLR4/NLRP3 signaling pathway, Bioengineered 13 (2022) 11684–11693.

[31] K. Wang, S. Hu, B. Wang, et al., Genistein protects intervertebral discs from degeneration via Nrf2-mediated antioxidant defense system: An *in vitro* and *in vivo* study, J. Cell. Physiol. 234 (2019) 16348–16356.

[32] M. Li, X. Yu, X. Chen, et al., Genkwanin alleviates intervertebral disc degeneration via regulating ITGA2/PI3K/AKT pathway and inhibiting apoptosis and senescence, Int. Immunopharmacol. 133 (2024), 112101.

[33] L. Yu, Y. Hao, Z. Ren, et al., Ginsenoside Rg1 relieves rat intervertebral disc degeneration and inhibits IL-1β-induced nucleus pulposus cell apoptosis and inflammation via NF-κB signaling pathway, Vitro Cell. Dev. Biol. Anim. 60 (2024) 287–299.

[34] L. Yu, Y. Hao, C. Peng, et al., Effect of Ginsenoside Rg1 on the intervertebral disc degeneration rats and the degenerative pulposus cells and its mechanism, Biomed. Pharmacother. 123 (2020), 109738.

[35] J. Chen, B. Zhang, L. Wu, et al., Ginsenoside Rg3 exhibits anti-catabolic and anti-apoptotic effects in IL-1β treated human disc nucleus pulposus cells and in a rat model of disc degeneration by inactivating the MAPK pathway, Cell. Mol. Biol. 70 (2024) 233–238.

[36] X. Liu, J. Zhuang, D. Wang, et al., Glycyrrhizin suppresses inflammation and cell apoptosis by inhibition of HMGB1 via p38/p-JUK signaling pathway in attenuating intervertebral disc degeneration, Am. J. Transl. Res. 11 (2019) 5105–5113.

[37] B. Ogunlade, O.P. Fidelis, S.A. Adelakun, et al., Grape seed extract inhibits nucleus pulposus cell apoptosis and attenuates annular puncture induced intervertebral disc degeneration in rabbit model, Anat. Cell Biol. 53 (2020) 313–324.

[38] X. Zhu, S. Liu, Z. Cao, et al., Higenamine mitigates interleukin-1β-induced human nucleus pulposus cell apoptosis by ROS-mediated PI3K/Akt signaling, Mol. Cell. Biochem. 476 (2021) 3889–3897.

[39] X. Bai, W. Ding, S. Yang, et al., Higenamine inhibits IL-1β-induced inflammation in human nucleus pulposus cells, Biosci. Rep. 39 (2019), BSR20190857.

[40] P. Tang, J. Gu, Z. Xie, et al., Honokiol alleviates the degeneration of intervertebral disc via suppressing the activation of TXNIP-NLRP3 inflammasome signal pathway, Free. Radic. Biol. Med. 120 (2018) 368–379.

[41] S. Yang, W. Liao, Hydroxysafflor yellow A attenuates oxidative stress injury-induced apoptosis in the nucleus pulposus cell line and regulates extracellular matrix balance via CA XII, Exp. Ther. Med. 23 (2021), 182.

[42] T. Xie, J. Yuan, L. Mei, et al., Hyperoside ameliorates TNF-α-induced inflammation, ECM degradation and ER stress-mediated apoptosis via the SIRT1/NF-κB and Nrf2/ARE signaling pathways *in vitro*, Mol. Med. Rep. 26 (2022), 260.

[43] W. Hua, S. Li, R. Luo, et al., Icariin protects human nucleus pulposus cells from hydrogen peroxide-induced mitochondria-mediated apoptosis by activating nuclear factor erythroid 2-related factor 2, Biochim. Biophys. Acta BBA Mol. Basis Dis. 1866 (2020), 165575.

[44] G. Zhang, Y. Liao, H. Yang, et al., Irigenin reduces the expression of caspase-3 and matrix metalloproteinases, thus suppressing apoptosis and extracellular matrix degradation in TNF-α-stimulated nucleus pulposus cells, Chem. Biol. Interact. 349 (2021), 109681.

[45] Y. Huang, J. Sun, S. Li, et al., Isoliquiritigenin mitigates intervertebral disc degeneration induced by oxidative stress and mitochondrial impairment through a PPARγ-dependent pathway, Free. Radic. Biol. Med. 225 (2024) 98–111.

[46] X. Wang, Y. Tan, F. Liu, et al., Pharmacological network analysis of the functions and mechanism of kaempferol from Du Zhong in intervertebral disc degeneration (IDD), J. Orthop. Transl. 39 (2023) 135–146.

[47] D. Wang, H. Qu, H. Kang, et al., Kukoamine A attenuates lipopolysaccharide-induced apoptosis, extracellular matrix degradation, and inflammation in nucleus pulposus cells by activating the P13K/Akt pathway, Bioengineered 13 (2022) 8772–8784.

[48] K. Wang, T. Chen, X. Ying, et al., Ligustilide alleviated IL-1β induced apoptosis and extracellular matrix degradation of nucleus pulposus cells and attenuates intervertebral disc degeneration *in vivo*, Int. Immunopharmacol. 69 (2019) 398–407.

[49] M. Guo, D. Wang, H. Liu, et al., Lupeol against high-glucose-induced apoptosis via enhancing the anti-oxidative stress in rabbit nucleus pulposus cells, Eur. Spine J. 27 (2018) 2609–2620.

[50] T. Xie, J. Yuan, L. Mei, et al., Luteolin suppresses TNF-α-induced inflammatory injury and senescence of nucleus pulposus cells via the Sirt6/NF-κB pathway, Exp. Ther. Med. 24 (2022), 469.

[51] J. Lin, J. Chen, Z. Zhang, et al., Luteoloside inhibits IL-1β-induced apoptosis and catabolism in nucleus pulposus cells and ameliorates intervertebral disk degeneration, Front. Pharmacol. 10 (2019), 868.

[52] J. Chen, M. Bian, L. Pan, et al., α-Mangostin protects lipopolysaccharide-stimulated nucleus pulposus cells against NLRP3 inflammasome-mediated apoptosis via the NF-κB pathway, J. Appl. Toxicol. 42 (2022) 1467–1476.

[53] M. Yao, J. Zhang, Z. Li, et al., Marein protects human nucleus pulposus cells against high glucose-induced injury and extracellular matrix degradation at least partly by inhibition of ROS/NF-κB pathway, Int. Immunopharmacol. 80 (2020), 106126.

[54] L. Nan, F. Wang, D. Ran, et al., Naringin alleviates H2O2-induced apoptosis via the PI3K/Akt pathway in rat nucleus pulposus-derived mesenchymal stem cells, Connect. Tissue Res. 61 (2020) 554–567.

[55] Y. Fang, J. Hu, Y. Zou, et al., Neochlorogenic acid combined with bone marrow mesenchymal stem cells encapsulated into GelMA hydrogel for transplantation to repair intervertebral disk degeneration, Biomacromolecules 25 (2024) 729–740.

[56] T. Xie, R. Pan, W. Huang, et al., Myricetin alleviates H2O2-induced senescence and apoptosis in rat nucleus pulposus-derived mesenchymal stem cells, Folia Histochem Cytobiol. 61 (2023) 98–108.

[57] Z. Zhang, J. Wu, C. Teng, et al., Orientin downregulating oxidative stress-mediated endoplasmic reticulum stress and mitochondrial dysfunction through AMPK/SIRT1 pathway in rat nucleus pulposus cells *in vitro* and attenuated intervertebral disc degeneration *in vivo*, Apoptosis 27 (2022) 1031–1048.

[58] H. Wang, Y. Ding, W. Zhang, et al., Oxymatrine liposomes for intervertebral disc treatment: Formulation, *in vitro* and vivo assessments, Drug Des. Dev. Ther. 14 (2020) 921–931.

[59] D. Guo, M. Yu, H. Guo, et al., Panax notoginseng saponins inhibits oxidative stress- induced human nucleus pulposus cell apoptosis and delays disc degeneration *in vivo* and *in vitro*, J. Ethnopharmacol. 319 (2024), 117166.

[60] E. Chen, M. Li, Z. Liao, et al., Phillyrin reduces ROS production to alleviate the progression of intervertebral disc degeneration by inhibiting NF-κB pathway, J. Orthop. Surg. Res. 19 (2024), 308.

[61] H. Chen, M. Liu, G. Zhang, et al., Proanthocyanidins inhibit the apoptosis and aging of nucleus pulposus cells through the PI3K/Akt pathway delaying intervertebral disc degeneration, Connect. Tissue Res. 63 (2022) 650–662.

[62] Y. Zou, Q. Zhang, Q. Zhang, et al., Procyanidin B2 alleviates oxidative stress-induced nucleus pulposus cells apoptosis through upregulating Nrf2 via PI3K–Akt pathway, J. Orthop. Res. 41 (2023) 1555–1564.

[63] H. Tang, S. Zhang, X. Lu, et al., Effects of puerarin on the intervertebral disc degeneration and biological characteristics of nucleus pulposus cells, Pharm. Biol. 61 (2023) 12–22.

[64] Z. Yu, X. Wang, Y. Hu, et al., Quercetin alleviates hyperglycemic-generated endoplasmic reticulum stress-contacted apoptosis of rat nucleus pulposus cells, Balkan Med. J. 42 (2025) 27–36.

[65] H. Kim, C. Yeo, J.Y. Hong, et al., Raphanus sativus linne protects human nucleus pulposus cells against H2O2-induced damage by inhibiting TREM2, Biology 13 (2024), 602.

[66] C. Wu, J. Ge, M. Yang, et al., Resveratrol protects human nucleus pulposus cells from degeneration by blocking IL-6/JAK/STAT3 pathway, Eur. J. Med. Res. 26 (2021), 81.

[67] X. Bai, X. Guo, F. Zhang, et al., Resveratrol combined with 17β-estradiol prevents IL-1β induced apoptosis in human nucleus pulposus via the PI3K/AKT/mtor and PI3K/AKT/GSK-3β pathway, J. Investig. Surg. 34 (2021) 904–911.

[68] J. Zhou, Q. Liu, Z. Yang, et al., Rutin maintains redox balance to relieve oxidative stress induced by TBHP in nucleus pulposus cells, Vitro Cell. Dev. Biol. Anim. 57 (2021) 448–456.

[69] J. Hu, C. Li, S. Jin, et al., Salvianolic acid B combined with bone marrow mesenchymal stem cells piggybacked on HAMA hydrogel re-transplantation improves intervertebral disc degeneration, Front. Bioeng. Biotechnol. 10 (2022), 950625.

[70] J. Huang, X. Zheng, J. Lin, et al., Sinapic acid inhibits IL-1β-induced apoptosis and catabolism in nucleus pulposus cells and ameliorates intervertebral disk degeneration, J. Inflamm. Res. 13 (2020) 883–895.

[71] G. Lu, C. Zhang, K. Li, et al., Sinomenine ameliorates IL-1β-induced intervertebral disc degeneration in rats through suppressing inflammation and oxidative stress via Keap1/Nrf2/NF-κB signaling pathways, J. Inflamm. Res. 16 (2023) 4777–4791.

[72] X. Xu, D. Wang, C. Zheng, et al., Progerin accumulation in nucleus pulposus cells impairs mitochondrial function and induces intervertebral disc degeneration and therapeutic effects of sulforaphane, Theranostics 9 (2019) 2252–2267.

[73] W. Qi, D. Ren, P. Wang, et al., Upregulation of Sirt1 by tyrosol suppresses apoptosis and inflammation and modulates extracellular matrix remodeling in interleukin-1β-stimulated human nucleus pulposus cells through activation of PI3K/Akt pathway, Int. Immunopharmacol. 88 (2020), 106904.

[74] C. Zhang, Z. Li, L. Li, et al., Achyranthoside D (AD) improve intervertebral disc degeneration through affect the autophagy and the activation of PI3K/Akt/mTOR pathway, J. Orthop. Surg. 30 (2022), 10225536221135474.

[75] J. Liu, D. Lin, J. Lu, Andrographolide influences IDD cell autophagy and oxidative stress undermechanical pressure via miR-9/FoxO3/PINK1/Parkin molecular axis, Cell. Mol. Biol. 70 (2024) 192–198.

[76] C. Xie, Y. Shi, Z. Chen, et al., Apigenin alleviates intervertebral disc degeneration via restoring autophagy flux in nucleus pulposus cells, Front. Cell Dev. Biol. 9 (2022), 787278.

[77] T. Xu, H. Zhao, J. Li, et al., Apigetrin alleviates intervertebral disk degeneration by regulating nucleus pulposus cell autophagy, Jor Spine 7 (2024), e1325.

[78] Y. Chen, Z. Zheng, J. Wang, et al., Berberine suppresses apoptosis and extracellular matrix (ECM) degradation in nucleus pulposus cells and ameliorates disc degeneration in a rodent model, Int. J. Biol. Sci. 14 (2018) 682–692.

[79] R. Luo, Z. Liao, Y. Song, et al., Berberine ameliorates oxidative stress-induced apoptosis by modulating ER stress and autophagy in human nucleus pulposus cells, Life Sci. 228 (2019) 85–97.

[80] L. Xiao, B. Ding, J. Gao, et al., Curcumin prevents tension-induced endplate cartilage degeneration by enhancing autophagy, Life Sci. 378 (2020), 118213.

[81] C. Wan, S. Liu, L. Zhao, et al., Curcumin protects rat endplate chondrocytes against IL-1β-induced apoptosis via Bcl-2/Bax regulation, J. Mol. Histol. 56 (2025), 111.

[82] X. Bai, M. Jiang, J. Wang, et al., Cyanidin attenuates the apoptosis of rat nucleus pulposus cells and the degeneration of intervertebral disc via the JAK2/STAT3 signal pathway *in vitro* and *in vivo*, Pharm. Biol. 60 (2022) 427–436.

[83] L. Nan, F. Wang, Y. Liu, et al., 6-gingerol protects nucleus pulposus-derived mesenchymal stem cells from oxidative injury by activating autophagy, World J. Stem Cells 12 (2020) 1603–1622.

[84] H. Yu, Y. Teng, J. Ge, et al., Isoginkgetin-loaded reactive oxygen species scavenging nanoparticles ameliorate intervertebral disc degeneration via enhancing autophagy in nucleus pulposus cells, J. Nanobiotechnol. 21 (2023), 99.

[85] Z. Wang, J. Ma, Y. Sun, et al., Isorhapontigenin delays senescence and matrix degradation of nucleus pulposus cells via PI3K/AKT/mTOR-mediated autophagy pathway *in vitro* and alleviates intervertebral disc degeneration *in vivo*, Int. Immunopharmacol. 139 (2024), 112717.

[86] Y. Lu, L. Zhou, S. He, et al., Lycopene alleviates disc degeneration under oxidative stress through the Nrf2 signaling pathway, Mol. Cell. Probes 51 (2020), 101559.

[87] F. Guo, Y. Zou, Y. Zheng, Moracin M inhibits lipopolysaccharide-induced inflammatory responses in nucleus pulposus cells via regulating PI3K/Akt/mTOR phosphorylation, Int. Immunopharmacol. 58 (2018) 80–86.

[88] T. Mao, J. Fan, Myricetin restores autophagy to attenuate lumbar intervertebral disk degeneration via negative regulation of the JAK2/STAT3 pathway, Biochem. Genet. 63 (2025) 2743–2759.

[89] Z. Zhang, C. Wang, J. Lin, et al., Therapeutic potential of naringin for intervertebral disc degeneration: Involvement of autophagy against oxidative stress-induced apoptosis in nucleus pulposus cells, Am. J. Chin. Med. 46 (2018) 1561–1580.

[90] R. Lu, H. Xu, X. Deng, et al., Physalin A alleviates intervertebral disc degeneration via anti-inflammatory and anti-fibrotic effects, J. Orthop. Transl. 39 (2023) 74–87.

[91] W. Hua, L. Xie, C. Dong, et al., Procyanidin C1 ameliorates acidic pH stress induced nucleus pulposus degeneration through SIRT3/FOXO3- mediated mitochondrial dynamics, J. Transl. Med. 22 (2024), 1071.

[92] D. Wang, X. He, D. Wang, et al., Quercetin suppresses apoptosis and attenuates intervertebral disc degeneration via the SIRT1-autophagy pathway, Front. Cell Dev. Biol. 8 (2020), 613006.

[93] B. Zhang, L. Xu, N. Zhuo, et al., Resveratrol protects against mitochondrial dysfunction through autophagy activation in human nucleus pulposus cells, Biochem. Biophys. Res. Commun. 493 (2017) 373–381.

[94] S. Hu, Y. Zou, Y. Jiang, et al., Scutellarin-mediated autophagy activates exosome release of rat nucleus pulposus cells by positively regulating Rab8a via the PI3K/PTEN/Akt pathway, Cell Biol. Int. 46 (2022) 1588–1603.

[95] Z. Gao, Y. Lin, P. Zhang, et al., Sinomenine ameliorates intervertebral disc degeneration via inhibition of apoptosis and autophagy *in vitro* and *in vivo*, Am. J. Transl. Res. 11 (2019) 5956–5966.

[96] S. Cai, R. Ding, H. Zhang, et al., Self-healing hydrogels loaded with Spatholobi Caulis alleviate disc degeneration by promoting autophagy in nucelus pulposus, Mater. Today Bio 29 (2024), 101323.

[97] J. Liu, P. Yu, F. Dai, et al., Tetrandrine reduces oxidative stress, apoptosis, and extracellular matrix degradation and improves intervertebral disc degeneration by inducing autophagy, Bioengineered 13 (2022) 3944–3957.

[98] Z. Niu, G. Tang, X. Wang, et al., Trigonochinene E promotes lysosomal biogenesis and enhances autophagy via TFEB/TFE3 in human degenerative NP cells against oxidative stress, Phytomedicine 112 (2023), 154720.

[99] J. Lin, J. Zhuge, X. Zheng, et al., Urolithin A-induced mitophagy suppresses apoptosis and attenuates intervertebral disc degeneration via the AMPK signaling pathway, Free. Radic. Biol. Med. 150 (2020) 109–119.

[100] G. Yang, X. Liu, X. Jing, et al., Astaxanthin suppresses oxidative stress and calcification in vertebral cartilage endplate via activating Nrf-2/HO-1 signaling pathway, Int. Immunopharmacol. 119 (2023), 110159.

[101] P. Zhang, K. Rong, J. Guo, et al., Cynarin alleviates intervertebral disc degeneration via protecting nucleus pulposus cells from ferroptosis, Biomed. Pharmacother. 165 (2023), 115252.

[102] C. Li, Y. Zhang, Y. Deng, et al., Fisetin suppresses ferroptosis through Nrf2 and attenuates intervertebral disc degeneration in rats, Eur. J. Pharmacol. 964 (2024), 176298.

[103] Z. Zhu, Z. Huang, C. Zhang, et al., Gallic acid protects intervertebral disc cells from ferroptosis and alleviates intervertebral disc degeneration by regulating key factors of oxidative stress, Front. Pharmacol. 16 (2025), 1501725.

[104] J. Zhu, R. Sun, C. Yan, et al., Hesperidin mitigates oxidative stress-induced ferroptosis in nucleus pulposus cells via Nrf2/NF-κB axis to protect intervertebral disc from degeneration, Cell Cycle 22 (2023) 1196–1214.

[105] Y. Shao, L. Sun, G. Yang, et al., Icariin protects vertebral endplate chondrocytes against apoptosis and degeneration via activating Nrf-2/HO-1 pathway, Front. Pharmacol. 13 (2022), 937502.

[106] H. Wang, X. Liu, H. Yang, et al., Activation of the Nrf-2 pathway by pinocembrin safeguards vertebral endplate chondrocytes against apoptosis and degeneration caused by oxidative stress, Life Sci. 333 (2023), 122162.

[107] Z. Liu, X. Li, J. Pan, et al., Oxidized low-density lipoprotein induced ferroptosis in nucleus pulposus cell contributes to intervertebral disc degeneration via LOX-1/NF-κB/NOX signal, Int. Immunopharmacol. 153 (2025), 114455.

[108] X. Bai, M. Yao, X. Zhu, et al., Baicalin suppresses interleukin-1β-induced apoptosis, inflammatory response, oxidative stress, and extracellular matrix degradation in human nucleus pulposus cells, Immunopharmacol. Immunotoxicol. 45 (2023) 433–442.

[109] B. Hu, S. Lin, S. Lin, et al., Ginkgetin alleviates intervertebral disc degeneration by inhibiting apoptosis, inflammation, and disturbance of extracellular matrix synthesis and catabolism via inactivation of NLRP3 inflammasome, Immunol. Investig. 52 (2023) 546–560.

[110] F. Zhao, Z. Guo, F. Hou, et al., Magnoflorine alleviates “M1” polarized macrophage-induced intervertebral disc degeneration through repressing the HMGB1/Myd88/NF-κB pathway and NLRP3 inflammasome, Front. Pharmacol. 12 (2021), 701087.

[111] Y. Gong, J. Qiu, T. Jiang, et al., Maltol ameliorates intervertebral disc degeneration through inhibiting PI3K/AKT/NF-κB pathway and regulating NLRP3 inflammasome-mediated pyroptosis, Inflammopharmacology 31 (2023) 369–384.

[112] Y. Zhou, Z. Chen, X. Yang, et al., Morin attenuates pyroptosis of nucleus pulposus cells and ameliorates intervertebral disc degeneration via inhibition of the TXNIP/NLRP3/Caspase-1/IL-1β signaling pathway, Biochem. Biophys. Res. Commun. 559 (2021) 106–112.

[113] J. Wang, X. Jing, X. Liu, et al., Naringin safeguards vertebral endplate chondrocytes from apoptosis and NLRP3 inflammasome activation through SIRT3-mediated mitophagy, Int. Immunopharmacol. 140 (2024), 112801.

[114] K. Tang, W. Su, C. Huang, et al., Notoginsenoside R1 suppresses inflammatory response and the pyroptosis of nucleus pulposus cells via inactivating NF-κB/NLRP3 pathways, Int. Immunopharmacol. 101 (2021), 107866.

[115] W. Dai, Z. Luo, Paeoniflorin inhibits pyroptosis of nucleus pulposus cells in an acidic environment and alleviates the degeneration of the intervertebral disc in rats, Cell. Signal. 91 (2022), 110243.

[116] J. Bao, W. Gao, W. Zhang, et al., Fibrin glue delivery system containing Rhein ameliorates intervertebral disc degeneration by anti-inflammatory efficacy, J. Orthop. Surg. Res. 18 (2023), 485.

[117] Z. Wang, P. Zhang, Y. Zhao, et al., Scutellarin protects against mitochondrial reactive oxygen species-dependent NLRP3 inflammasome activation to attenuate intervertebral disc degeneration, Front. Bioeng. Biotechnol. 10 (2022), 883118.

[118] P. Yu, Z. Ma, H. Jiang, et al., Sinapine thiocyanate alleviates intervertebral disc degeneration by not regulating JAK1/STAT3/NLRP3 signal pathway, Adv. Clin. Exp. Med. 33 (2024) 965–977.
